# Comparing tuberculosis management under public and private healthcare providers: Victoria, Australia, 2002**–**2015

**DOI:** 10.1186/s12879-017-2421-x

**Published:** 2017-05-03

**Authors:** Katie D. Dale, Ee Laine Tay, James M. Trauer, Peter G. Trevan, Justin T. Denholm

**Affiliations:** 1 Victorian Tuberculosis Program, The Peter Doherty Institute for Infection and Immunity, Victoria, Australia; 2 Department of Health and Human Services, Victoria, Australia; 3 School of Public Health and Preventive Medicine, Monash University, Victoria, Australia; 4 Department of Microbiology and Immunology, The University of Melbourne, Victoria, Australia

**Keywords:** Private sector, Public sector, Time-to-treatment, Delayed diagnosis, Patient care

## Abstract

**Background:**

Private healthcare providers are important to tuberculosis (TB) management globally, although internationally there are reports of suboptimal management and disparities in treatment commencement in the private sector. We compared the management of TB patients receiving private versus public healthcare in Victoria, an industrialised setting with low tuberculosis (TB) incidence.

**Methods:**

Retrospective cohort study: 2002–2015. Private healthcare provision was included as an independent variable in several multivariate logistic and Cox proportional hazard regression models that assessed a range of outcome variables, encompassing treatment commencement delays, management and treatment outcomes.

**Results:**

Of 5106 patients, 275 (5.4%) exclusively saw private providers, and 4714 (92.32%) public. Private care was associated with a shorter delay to presentation (HR 1.36, *p* = 0.065, 95% CI 1.02–2.00). Private patients were less likely to have genotypic testing (OR 0.66, *p* = 0.009, 95% CI 0.48–0.90), those with pulmonary involvement were less likely to have a sputum smear (OR 0.52, *p* = 0.011, 95% CI 0.31–0.86) and provided samples were less likely to be positive (OR 0.54, *p* = 0.070, 95% CI 0.27–1.05). Private patients with extrapulmonary TB were less likely to have a smear sample (OR 0.7, 95% CI 0.48–0.90, *p* = 0.009) and radiological abnormalities (OR 0.71, *p* = 0.070, 95% CI 0.27–1.05). Treatment commencement delays from presentation were comparable for cases with pulmonary involvement and extrapulmonary TB, although public extrapulmonary TB patients received radiological examinations slightly earlier than private patients (HR 0.79, *p* = 0.043, 95% CI 0.63–0.99) and public patients with pulmonary involvement from high burden settings commenced treatment following an abnormal CXR more promptly than their private counterparts (HR 0.41, *p* = 0.011, 95% CI 0.21–0.81). Private patients were more likely to receive <4 first-line medications (OR 2.17, *p* = 0.001, 95% CI 1.36–3.46), but treatment outcomes were comparable between sectors.

**Conclusions:**

The differences we identified are likely to reflect differing case-mix as well as clinician practice. Sputum smear status was an important covariable in our analysis; with its addition we found no significant disparity in the health-system delay to treatment commencement between sectors. Our study highlights the importance of TB programs engaging with private providers, enabling comprehensive data collection that is necessary for thorough and true comparison of TB management and optimisation of care.

**Electronic supplementary material:**

The online version of this article (doi:10.1186/s12879-017-2421-x) contains supplementary material, which is available to authorized users.

## Background

Private healthcare providers are important to tuberculosis (TB) management globally, contributing an estimated 6–48% of all total case notifications in 2015 [1]. However, reports from a variety of contexts have suggested differences in the timely diagnosis and initiation of therapy between public and private sectors [2]. Longer delays have been identified in the private sector, including treatment delays (variably defined as the time from symptom onset to either smear test result, diagnosis or treatment commencement) [3–6] and health system delays (time from healthcare presentation to treatment initiation) [7–9]. Surveys of private providers have revealed TB patient management practices that fall short of recommended guidelines in various settings [10–16].

Despite these reports of suboptimal management and disparities in treatment initiation, there are very few studies that compare the care provided and treatment outcomes between private and public settings. A study in Vietnam found poorer management and treatment outcomes of patients treated in one semi-private clinic compared to patients treated by the national TB control program [17], but another in Nigeria reported comparable outcomes [18]. While it is acknowledged that the size, structure and function of private healthcare providers in health systems vary greatly between settings, to our knowledge there have been no studies that have investigated diagnostic delays, TB management and outcomes for patients receiving private versus public healthcare in any setting.

Victoria is Australia’s second most populous state with an estimated resident population of 5.97 million (December quarter 2015) people [19], universal health care and both a low incidence of TB (6.7/100,000 in 2013) [20] and low TB-related mortality [21]. TB patients are predominantly managed in the public sector, although some do attend private healthcare providers. The Victorian TB program (VTP) engages with both public and private providers and collects detailed data on all TB patients diagnosed in Victoria, including consistently recording whether care is provided through private or public services. All microbiological diagnoses of TB, regardless of healthcare sector, are managed centrally, with routine molecular testing conducted at a single statewide reference laboratory [22]. In the interest of ensuring that TB care is optimal in Victoria, we sought to compare the quality of healthcare received between public and private settings. Specifically, we aimed to investigate whether disparities in treatment initiation and TB management reported elsewhere in the world are present in Victoria, and to examine any effect on treatment outcomes.

## Methods

### Study population

The study population comprised patients diagnosed with active TB in Victoria and notified to the Department of Health & Human Services between 1/1/2002 and 31/12/2015. In Victoria, all medical practitioners and diagnostic laboratories are required to report patients with TB to health authorities under public health legislation [20]. All patients, regardless of their choice of health provider, are followed up by the VTP. Confirmation of TB diagnosis by culture and drug susceptibility testing is routinely performed where possible [23].

Australia has a publicly funded universal health insurance scheme (Medicare). TB management within public hospitals and clinics are provided free of charge to all TB patients in Victoria [24]. Patients that choose to attend private hospitals or physicians are frequently charged additional fees over and above the cost that is covered by Medicare or their private health insurers. Medications for the treatment of TB are provided free of charge for all patients by the VTP.

Patient records were extracted from an existing programmatic database (Public Health Event Surveillance System, PHESS), which contains detailed demographic information, laboratory results and patient notes including notifying medical practitioners and hospital identification numbers.

### Definitions

Sites of disease were categorised as cases with pulmonary involvement (excluding disseminated cases), disseminated and extrapulmonary manifestations (including lymph node TB). Any disease with two non-pulmonary, non-contiguous sites of disease was categorised as disseminated.

**Fig. 1:**
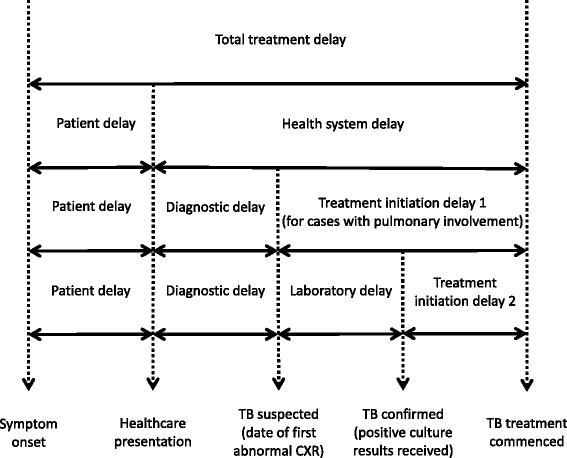
Conceptual illustration of definitions of delays (adapted from Van Wyk et al. 2011)

Figure 1 illustrates definitions of the various possible delays during TB management. Symptom onset, healthcare presentation dates, treatment commencement dates and dates of chest x-ray/CT-scan (CXR) were as specified on notification forms, or obtained by VTP nurses from clinicians, patients or medical records. Specimen collection dates and dates that test results were received were as recorded by laboratories.

### Statistical analyses

Wilcoxon-Mann-Whitney tests and Chi squared tests were used to compare demographic and clinical differences between public and private cohorts.

Private healthcare provision was included as an independent variable in univariate and multivariate logistic regression to analyse the investigations, symptoms, laboratory tests, treatment regimens and treatment outcomes. All independent variables with *P* < 0.25 in univariate analysis were considered in multivariate analysis [25].

Kaplan-Meier curves were used to illustrate various delays in diagnosis and treatment between public and private cohorts, and private healthcare provision was included as an independent variable in Cox proportional hazard analyses to assess these delays (Table 3). All independent variables with *P* < 0.25 in univariate (log-rank) analysis were considered in multivariate analysis [25]. Proportionality assumptions were assessed using Kaplan-Meier survival curves; by including time-dependent covariates in the model; and with Schoenfeld and scaled Schoenfeld residuals. Interactions were explored and likelihood-ratio tests, Akaike information criterions (AIC) and multiway cross tabulations were performed to guide which to include. No interactions causing variable combination frequencies of less than ten were included, except where noted.

In multivariate analyses, *p*-values below 0.05 were considered significant, although findings with *p*-values less than 0.10 are also discussed.

Analyses involving symptom onset data were only possible from 2012 onwards for pulmonary cases, due to limited prior data.

All data preparation and analysis was conducted using Microsoft Excel 2010 and Stata version 13.1 (Stata Corp., College Station, TX, USA).

## Results

Of 5106 TB patients, 275 (5.4%) were seen exclusively by private healthcare providers, and 4714 (92.3%) by public. Excluded from analysis were patients who moved from private to public healthcare during their care (*n* = 18, 0.4%), those who moved from public to private care (*n* = 2, 0.04%), those for whom the healthcare service provider was unable to be classified due to missing data, patient transfer overseas or death before healthcare presentation (*n* = 97, 1.9%). The characteristics of TB patients attending private and public healthcare providers, and their treatment outcomes, are listed in Table 1.

**Table 1:** Characteristics of tuberculosis cases notified in Victoria from 2002 to 2015

Variable	All notifications		Public		Private	
	*n* = 5106		*n* = 4714		*n* = 275	
	*n*	(%)^a^	*n*	(%)^a^	*n*	(%)^a^
Sex						
Female	2324	(45.5)	2120	(45.0)	141	(51.3)
Male	2782	(54.5)	2594	(55.0)	134	(48.7)
Age group, years						
< 18	365	(7.2)	362	(7.7)	1	(0.4)
18–65	3876	(83.1)	3587	(76.1)	206	(74.9)
≥ 65	865	(16.9)	765	(16.2)	68	(24.7)
Median age (years)	33	(IQR 25–53)	33	(IQR 25–52)	41	(IQR 30–64)
Place of birth						
Australia	543	(10.6)	466	(9.9)	45	(16.4)
Overseas born	4563	(89.4)	4248	(90.1)	230	(83.6)
Risk factors						
Substance abuse	82	(1.6)	74	(1.6)	1	(0.4)
Born in a high-burden country	3005	(58.9)	2797	(59.3)	156	(56.7)
Ever resided in an aged care facility	41	(0.8)	36	(0.8)	4	(1.5)
Indigenous	9	(0.2)	7	(0.1)	1	(0.4)
Household member or close contact with TB	904	(17.7)	852	(18.1)	34	(12.4)
Rural healthcare provider	101	(2.0)	94	(2.0)	6	(2.2)
Manifestation						
Pulmonary involvement	2605	(51.0)	2248	(47.7)	83	(30.2)
Disseminated	269	(5.3)	255	(5.4)	10	(3.6)
Extrapulmonary	2232	(43.7)	1993	(42.3)	182	(66.2)
Drug resistance^b^						
Fully sensitive	2978	(75.2)	2783	(75.9)	148	(71.2)
Multidrug-resistant TB (MDR-TB)^c^	71	(1.8)	67	(1.8)	3	(1.4)
Mono or poly-resistant, not MDR-TB	285	(7.2)	268	(7.3)	11	(5.3)
Not recorded	619	(15.6)	535	(14.6)	46	(22.1)
Median treatment duration in those completing treatment (days)	215	(IQR 184–289)	215	(IQR 184–288)	216	(IQR 185–281)
HIV-positive (2009–2015)	39/2739	(1.3)	39/2588	(10.0)	0/119	(28.6)

Definition of *abbreviations*: *TB* tuberculosis, *CXR* chest x-ray/CT scan, *IQR* interquartile range

^a^Except where stated

^b^Confined to culture-positive cases: All notifications = 3963 cases; Public = 3665 cases; Private = 208 cases

^c^Defined as resistance to both isoniazid and rifampicin at initial drug susceptibility testing.

There were several significant demographic and clinical differences between private and public patients. Those attending private health care were significantly older (*p* < 0.001), less likely to be born overseas (*p* = 0.001), less likely to have pulmonary involvement (*p* < 0.001) and more likely to have extrapulmonary TB (*p* < 0.001) (Table 1).

### Multivariate logistic regression

Table 2 presents results of private healthcare provision as an independent variable in univariate and multivariate logistic regression analyses of laboratory tests, results, treatment and outcomes. Full tables of results are provided in the Additional file 1: Tables S1–S21.

**Table 2:** Results for the independent variable ‘private healthcare provision’ in multivariate logistic regression analyses of laboratory tests, treatment regimens and outcomes among adult Victorian TB patients, 2002–2015

Outcome	Public patients: outcome present/total	Private patients: outcome present/total	Univariate analysis			Multivariate analysis^a^			
			OR	(95%CI)	*p*	aOR	(95%CI)	*P* value	Other independent variables
Investigations performed									
CXR^b^	2324/2466	74/83	0.50	(0.25–1.02)	0.058	0.56	(0.26–1.18)	0.125	a sx p s c h o I y^‡^
CXR - extrapulmonary patients	1747/1993	164/182	1.28	(0.78–2.13)	0.333	NA			a sx y^‡^
Smear sputum sample^b^	1937/2466	58/83	0.63	(0.39–1.02)	0.062	0.52	(0.31–0.86)	0.011	a^‡^ sx p^‡^ pe^‡^ c h o y^‡^
Bronchial wash sample^b sp.^	789/2466	35/83	1.55	(0.99–2.42)	0.053	1.49	(0.75–2.96)	0.252	p s f c^‡^ h o y^‡^ cx^‡^ sp.^‡^ c#h^‡^
Smear sample – extrapulmonary patients	1306/1993	96/182	0.59	(0.43–1.83)	0.001	0.54	(0.37–0.77)	0.001	a^‡^ sx p h o c y^‡^ cx^‡^
Genotypic TB testing^c^	2151/4518	93/268	0.58	(0.45–0.76)	<0.001	0.65	(0.47–0.89)	0.008	a pe^‡^ ly e d r p^‡^ h y^‡^ s o cx cx#m
Investigation results									
Abnormal first chest x-ray/CT scan^b^	2171/2271	69/70	3.18	(0.44–23.12)	0.253	NA			a sx pe^‡^ r^‡^ o y
Abnormal first chest x-ray/CT scan - extrapulmonary patients	655/1650	44/146	0.66	(0.45–0.95)	0.024	0.71	(0.48–1.04)	0.080	a^‡^ sx e^‡^ p c h o y^‡^
Cavitation	431/2156	10/69	0.68	(0.34–1.34)	0.262	NA			sx pe‡ r s h‡ y‡
Smear-positive sputum sample^b^	789/1937	19/58	0.71	(0.41–1.24)	0.225	0.54	(0.27–1.05)	0.070	pe^‡^ r^‡^ p s^‡^ h f cx^‡^ y^‡^
Smear positive – extrapulmonary patients	296/1093	34/87	1.73	(1.10–2.71)	0.017	1.51	(0.89–2.56)	0.132	e p h^‡^ y^‡^ cx^‡^
Positive genotypic test	1811/2151	79/93	1.00	(0.58–1.73)	0.998	NA			a^‡^ r cx pe ly^‡^ e^‡^ d^‡^ s o y^‡^
Culture-positive	3665/3968	208/229	0.82	(0.51–1.30)	0.398	NA			sx pe ly^‡^ e^‡^ d r h y cx
Presumptive diagnoses – not confirmed by culture or PCR.	839/4706	54/273	1.14	(0.84–1.54)	0.414	NA			a^‡^ sx pe ly^‡^ e^‡^ d r^‡^ s^‡^ f c h o y cx
Treatment commencement									
Prior to any positive test result or abnormal chest x-ray/CT scan	1053/4149	77/221	1.57	(1.18–2.09)	0.002	1.10	(0.80–1.51)	0.575	a sx pe ly^‡^ e^‡^ d^‡^ r p f c h o y^‡^ h#m^‡^
Following abnormal chest x-ray/CT scan, before any other results^b^	998/2135	17/48	0.42	(0.34–0.74)	0.003	0.43	(0.23–0.78)	0.006	a^‡^ r^‡^ p^‡^ s^‡^ c^‡^ y^‡^
Following positive culture^b sp^	327/2405	22/76	2.59	(1.56–4.31)	<0.001	1.22	(0.58–2.54)	0.604	a^‡^ sx pe p f c sp.^‡^
Following positive culture, extrapulmonary patients	305/1918	38/173	1.49	(1.02–2.18)	0.040	1.15	(0.72–1.84)	0.548	a sx e^a.^ r p s y cx
Treatment regimen and outcome									
Fewer than four first-line medications^d^	332/3996	29/208	1.79	(1.19–2.69)	0.005	2.17	(1.36–3.46)	0.001	a^‡^ sx^‡^ pe ly e^‡^ d p^‡^ f^‡^ c h^‡^ o y^‡^
Completed treatment	4043/4147	225/235	0.58	(0.30–1.12)	0.106	0.61	(0.31–1.21)	0.158	a sx pe^‡^ ly e^‡^ d p s^‡^ h y^‡^
Died before or during treatment	195/4288	17/244	1.57	(0.94–2.63)	0.084	1.16	(0.60–2.23)	0.652	a^‡^ sx^‡^ pe ly^‡^ e d^‡^ r p s^‡^ f^‡^ c h^‡^ o y

Definition of *abbreviations*: *TB* tuberculosis; *NA* not applicable, not included in multivariate analysis, private *p* > 0.25 in univariate analysis, *obs* observations, *lab* laboratory, *OR* odds ratio, *aOR* adjusted odds ratio, *IQR* interquartile range, # interaction term

^a^Variables considered in all univariate analyses – age (a) (five groups: 0–9 years; 10–17 years; 18–34 years; 35–64 years, ≥65 years); sex (sx); Manifestation (m) (pulmonary only, pulmonary plus other sites [pe], lymph node [ly], disseminated [d] or other extrapulmonary [e]); saw rural health provider (r); saw private health provider (p); history of substance abuse (s); ever resided in an aged care facility (f); household member or close contact with TB (c); born in high burden country (h); overseas born (o); year of notification (y) (groups: 2002–2005; 2006–2011; 2012–2015); First CXR results (cx) were also considered for all “Investigations performed” and “Investigation results” and “Treatment commencement” analyses except those regarding CXRs. Sputum smear results (sp) were included in several analyses where noted

^b^Only patients with pulmonary involvement included in the analysis

^c^Only patients with a specimen sample included in analysis

^d^Confined to patients that commenced treatment; began on treatment prior to culture results being available; and were not identified as a part of a screening or contact investigation

^‡^Independent variables included in multivariate analyses with *p*<0.05

In multivariate analysis, when compared to TB patients that attended public providers, patients who attended private providers were significantly less likely to have a genotypic investigation and were significantly less likely to have a sputum smear sample taken (for those with pulmonary involvement) and smear samples were less likely to be a positive. Private patients with extrapulmonary TB were significantly less likely to have a smear sample. Those with pulmonary involvement were significantly less likely to begin on treatment following an abnormal CXR and prior to other test results.

Patients who attended private healthcare providers were significantly more likely to receive fewer than four first-line medications, but there were no observed differences in treatment outcomes between public and privately treated patients.

### Delays and survival analysis

**Fig. 2:**
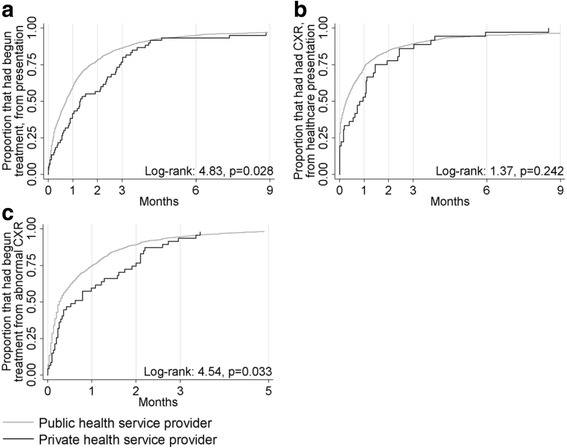
Kaplan-Meier curves for TB patients with pulmonary involvement in Victoria from 2002 to 2015. **a** Health system delay. **b** Diagnostic delay to first CXR. **c** Treatment initiation delay 1

**Fig. 3:**
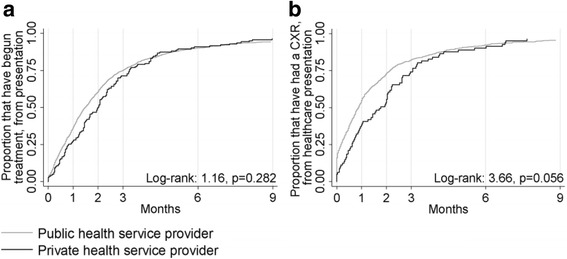
Kaplan-Meier curves for extrapulmonary TB patients in Victoria from 2002 to 2015. **a** Health system delay. **b** Diagnostic delay to first CXR

**Fig. 4:**
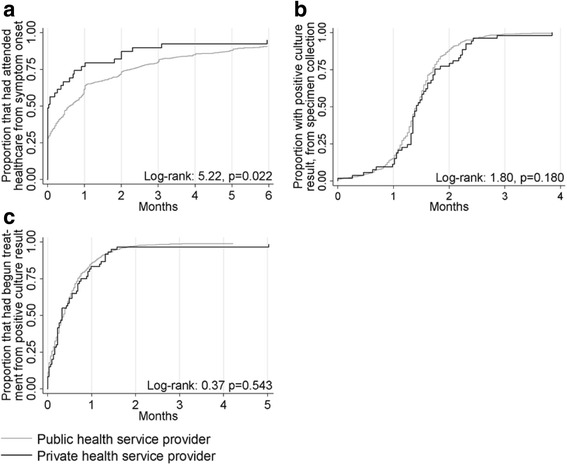
Kaplan-Meier curves for TB patients in Victoria. **a** Patient delay 2012–2015**. b** Laboratory delay 2011–2015**. c** Treatment initiation delay 2 2002-2015

Considering the median delays (Table 3), log-rank and Kaplan-Meier survival curves (Figs. 2, 3 and 4), private patients did not commence treatment as promptly as patients that attended public healthcare providers. The only disparities that remained in Cox proportional hazard analysis were that private patients attended healthcare sooner after symptom onset than public patients, private extrapulmonary TB patients received radiological examinations less promptly than public patients and a significant interaction in the model of treatment initiation delay 1 indicated that private patients born in a high burden country with pulmonary involvement (*n* = 20) were not initiated on treatment as quickly as their public counterparts following an abnormal CXR (Table 3). Full tables of results are provided in the Additional file 1: Tables S22–S29.

**Table 3:** Results for the independent variable ‘private healthcare provision’ in Cox proportional hazard model analyses of various time periods between symptom onset, healthcare presentation, investigations and treatment commencement among adult Victorian TB adult patients, 2002–2015

Time period outcome	Private sample	Private healthcare provision	Public healthcare provision	Results of Cox regression analysis with inclusion of private healthcare as binary exposure variable Multivariate survival analysis^a^				
		Median (IQR) (days)	Median (IQR) (days)	Number of obs	HR	(95%CI)	*P* value	Other independent variables
Patients with pulmonary involvement								
Health system delay ^cx sp^	59	40 (17–90)	22 (6–52)	1399	0.81	(0.58–1.12)	0.195	a^‡^ sx^‡^ pe p h^‡^ o^‡^ r sp.^‡^
Diagnostic delay - presentation to first chest x-ray/CT scan	35	29 (5–59)	9 (0–32)	1216	0.84	(0.60–1.18)	0.307	sx^**‡**^ pe r p h^**‡**^ o y^**‡**^
Treatment initiation delay 1 ^sp^	83	19 (7–64)	10 (3–34)	1409	1.35	(0.82–2.21)	0.235	a^**‡**^ p s^**‡**^ c h o y sp.^**‡**^ h#p^**‡**^ a#o^**‡**^ a#sp.^**‡**^ (this interaction term contained no smear positive in the youngest age-group)
Extrapulmonary patients								
Health system delay ^cx^	143	61 (25–99)	46 (20–91)	1504	NA			sx e f‡ c‡ y‡ cx cx#m‡
Diagnostic delay - presentation to first chest x-ray/CT scan	81	54 (19–93)	27 (6–65)	1080	0.79	(0.63–0.99)	0.043	s‡ sx p‡ y‡
All patients								
Patient delay^b^	39	1 (0–28)	18 (0–68)	871	1.36	(1.02–2.00)	0.065	sx pe ly e d p c o
Laboratory delay - specimen collection to culture result^c^	53	43 (40–53)	43 (35–52)	1380	0.90	(0.68–1.20)	0.474	sx pe ly‡ e‡ d‡ r s‡ p c o c#sx‡ sx#o‡
Treatment initiation delay 2	60	10 (5–25)	11 (3–21)	577	NA			a sx y‡ sm‡

Definition of *abbreviations*: *TB* tuberculosis, *NA* not applicable, private *p* > 0.25 in univariate analysis, *obs* observations, *IQR* interquartile range, *HR* hazard ratio, *#* interaction operator

^a^Variables considered in univariate analysis – age (a) (five groups: 0–9 years; 10–17 years; 18–34 years; 35–64 years, ≥65 years); sex (sx); manifestation (m) (categories: pulmonary only, pulmonary plus other sites [pe], lymph node [ly], disseminated [d] or other extrapulmonary [e]); saw rural health provider (r); saw private health provider (p); has a history of substance abuse (s); has ever resided in an aged care facility (f); household member or close contact with TB (c); born in high burden country (h); overseas born (o); year of notification (y) (groups: 2002–2005; 2006–2011; 2012–2015);. First CXR results (cx) and sputum smear results (sp) and smear results (sm) were also considered where noted

^b^Patient delay could only be considered from 2012 to 2015 due to small sample numbers in prior years.

^c^Laboratory delay could only be considered from 2011 to 2015 due to year of notification variable not being proportional

^‡^Independent variables included in multivariate analyses with *p*<0.05

## Discussion

In our setting, there were differences in the care received by TB patients in private compared to public healthcare settings that are likely to reflect both differences in clinician practice and the case-mix managed. Private physicians were less likely to perform genotypic testing, take a sputum sample, and less likely to prescribe all four first-line medications. Patients attending private healthcare did so sooner after symptom onset, were less likely to have positive sputum specimens and certain groups did not receive assessments or commence treatment as promptly as public patients. Despite these disparities, treatment outcomes were comparable and no significant differences in health system delays to treatment commencement were found in Cox proportional hazard analyses.

The ability to distinguish patient delay from health system delays allowed us to demonstrate that in our setting public and private patients exhibited different health-seeking behaviour, with private patients attending sooner after symptom onset. Although it has been proposed that seeking medical care more promptly may indicate more severe disease and symptoms [26], our results were consistent with studies showing a negative association between symptom duration and smear-positivity [27, 28]. In our cohort, private patients with pulmonary involvement were less likely to have a smear-positive sputum sample than public patients, and extrapulmonary private patients were less likely to have an abnormal CXR, suggesting that private patients may present to healthcare with less severe disease. The degree to which the disparity in health-seeking behaviour reflects barriers that public patients experienced in accessing their care is unknown, and collection of more detailed qualitative information on health-seeking behaviour would be valuable.

Following healthcare attendance, several of our findings suggested that certain groups of private patients do not receive assessments or commence treatment as promptly as public patients. While it is possible these findings are due to differences in clinician practice, it may also reflect differing disease severity of the cohorts presenting to each provider type. For example, the lower likelihood of sputum examination in private patients may reflect a decreased ability to expectorate rather than clinician practice. Similarly, the fact that private extrapulmonary patients didn’t receive radiological examinations as promptly and certain groups took longer to commence treatment following an abnormal radiological result may be consistent with either 1) avoidable diagnostic delays, 2) logistical issues related to access to radiological and/or treatment, or 3) generally less severe presentations posing little risk of significant morbidity and disease transmission or greater diagnostic difficulty. While contributions from the first two possibilities cannot be discounted, the latter explanation is supported by investigation results among private patients indicating less severe disease and the fact that no significant health system delay was present overall, and treatment outcomes were comparable. From a public health perspective, the patterns of earlier presentation, less frequent smear-positivity and less severe disease also suggest a lower risk of transmission of infection.

Despite this, two of our findings, necessitate action due to the increasing incidence of multi-drug resistant TB (MDR-TB) in our setting, namely the less frequent use of genomic diagnostics and of a full complement of first-line drugs in private settings [20]. The VTP engages closely with both public and private physicians, so educational engagement will be possible across the sector to ensure improved uptake of genotypic testing and appropriate treatment regimens.

After adjustment for covariables (listed in the comments section of Table 3), we found no significant difference in health system or laboratory delays between public and private patients using Cox proportional hazard analysis. Similar analyses in other settings have revealed treatment delays by private providers and have proposed that these may be due to “deficiencies” [3], poorer knowledge regarding TB management [7, 8] or a lack of “effective diagnostic tools and follow-up routines” [2]. Although these factors may well be contributing, most previous studies did not consider important covariables, such as sputum smear status, which have previously been associated with health system delays [28] and that we found to be an important confounder in analyses of treatment commencement. A physician’s index of suspicion and their decision to commence treatment is inevitably influenced by their own knowledge, experience and biases, but also by factors beyond their control, including patient characteristics, manifestation, severity of disease and investigation results. These factors differed between public and private patients in our setting, and had an important influence on our results. Understanding the effect of such covariables is therefore important in any analysis of TB management.

The majority of studies comparing public and private settings have been performed in middle to high incidence settings that differ from ours. Factors associated with diagnostic and treatment delays may differ due to differing health systems, types of practitioners and health-seeking behaviour [4], so results are likely to be context-specific. In our setting, all TB patients, both public and private, are supervised and monitored by the VTP and TB medication is free– factors that have long been identified as leading to successful implementation of TB management [29]. The VTP has improved engagement with both public and private sectors over time, which allows comprehensive data collection, detailed analysis of care and effective feedback of results to practitioners. Our analyses showed that programmatic indicators such as treatment completion improved in our setting over the period of observation. In other settings, including many that have high TB burdens and growing private health sectors [30], we recognise that such engagement may be absent, data may be unavailable, and comprehensive and meaningful assessment of healthcare may be impossible. Fostering engagement between public and private healthcare sectors should therefore be the first priority in such settings. As has been noted, “private sector involvement might not be a bad thing in itself, but… public resources must be more effectively deployed for capturing and curating data of public interest from the private sector” [30].

As a well-resourced setting, we are privileged to have a large, well-curated dataset, and can provide a comprehensive analysis of TB management in both sectors, including treatment outcomes. However, we acknowledge the uncertainty inherent in any statistical analyses and the limitations of making multiple statistical comparisons, and urge caution in the interpretation of presented differences. Our study is also limited by the use of retrospective surveillance data and therefore subject to the influence of factors such as changes in data collection practices over time. Furthermore, analyses of some aspects of care were impossible due to small samples of private patients. We were also unable to include several covariables in analyses that have been shown to affect health system delays in some settings, including comorbidities [31] and education level [32]. However, the addition of comorbidities may further moderate disparities in diagnostic delay in our study, given the older age of private patients [33]. Further exploration regarding health-seeking behaviour and access to care, and the inclusion of outcomes such as relapse and the extent of onward transmission are planned.

## Conclusions

We found small differences between public and private sectors in approach to care that are likely to reflect a differing case-mix as well as differing clinician practice. With the addition of covariables such as sputum smear status in Cox regression no significant differences in health system delays to treatment commencement were found, and treatment outcomes were comparable between public and private patients. The private sector is a significant provider of TB care globally [1]. In many countries, significant progress has been made in capturing notifications from the private sector, although considerable work is still required to foster communication and cooperation between sectors. Our study highlights the importance of having a TB program that engages well with both public and private providers, enabling comprehensive data collection that is necessary for thorough and true comparison of TB management and optimisation of care.

## Additional file

Additional file 1:
**Tables S1**–**S29**. Univariate and multivariate results from all logistic regression and Cox proportional hazard regression analyses referred to in Tables 2 and 3. (DOCX 161 kb)

## Abbreviations

aOR: Adjusted odds ratio
CXR: Chest x-ray/CT-scan
HR: Hazard ratio
IQR: Interquartile range
lab: Laboratory
MDR-TB: Multi-drug resistant TB
NA: Not applicable, not included in multivariate analysis, private *p* > 0.25 in univariate analysis
obs: Observations
OR: Odds ratio
PHESS: Public Health Event Surveillance System
TB: Tuberculosis
VTP: The Victorian TB program
