# Supplementary material for: Comparing tuberculosis management under public and private healthcare providers: Victoria, Australia, 2002–2015
Source: BMC Infect Dis. 2017 May 3;17:324. doi: 10.1186/s12879-017-2421-x (PMC5415803; doi:10.1186/s12879-017-2421-x)
Supplement: Additional file 1: — Tables S1–S29. Univariate and multivariate results from all logistic regression and Cox proportional hazard regression analyses referred to in Tables 2 and 3. (DOCX 161 kb) [file 12879_2017_2421_MOESM1_ESM.docx]

# Appendices

**Investigations undertaken (from Table 2)**

**Table S1: Univariate and multivariate logistic regression analyses of factors associated with whether a chest-x-ray/CT scan was performed among Victorian TB patients with pulmonary involvement, 2002-2015.**

|  | **Univariate analysis** | | |  | **Multivariate analysis** | | |
| --- | --- | --- | --- | --- | --- | --- | --- |
| **Variable** | **OR** | **(95%CI)** | **P value** |  | **OR** | **(95%CI)** | **P value** |
| Sex: Males versus females | 0.81 | (0.58-1.13) | 0.216 |  | 0.86 | (0.60-1.22) | 0.388 |
| Age, years (versus 0-9 years) |  |  |  |  |  |  |  |
| 10-17 | 1.62 | (0.52-4.99) | 0.404 |  | 2.05 | (0.64-6.62) | 0.229 |
| 18-34 | 1.85 | (0.88-3.86) | 0.103 |  | 2.43 | (1.02-5.82) | 0.045 |
| 35-64 | 1.15 | (0.55-2.42) | 0.704 |  | 1.58 | (0.66-3.76) | 0.302 |
| ≥65 | 0.74 | (0.35-1.55) | 0.428 |  | 1.21 | (0.51-2.85) | 0.666 |
| Year of notification (versus 2002-2005) |  |  |  |  |  |  |  |
| 2006-2011 | 1.51 | (1.08-2.12) | 0.017 |  | 1.45 | (1.01-2.07) | 0.044 |
| 2012-2015 | 11.24 | (6.16-20.51) | <0.001 |  | 11.04 | (5.89-20.70) | <0.001 |
| Healthcare provider |  |  |  |  |  |  |  |
| Private | 0.50 | (0.25-1.02) | 0.058 |  | 0.56 | (0.26-1.18) | 0.125 |
| Rural | 0.72 | (0.29-1.83) | 0.494 |  |  |  |  |
| Risk factors considered |  |  |  |  |  |  |  |
| Overseas born | 1.49 | (1.00-2.23) | 0.048 |  | 1.11 | (0.66-1.88) | 0.690 |
| Born in a high burden country | 1.36 | (0.99-1.86) | 0.059 |  | 1.02 | (0.69-1.51) | 0.913 |
| Household member of close contact with TB | 1.36 | (0.90-2.07) | 0.145 |  | 1.44 | (0.88-2.35) | 0.143 |
| Ever resided in an aged care facility | 1.82 | (0.25-13.46) | 0.559 |  |  |  |  |
| History of substance abuse | 0.38 | (0.18-0.79) | 0.009 |  | 0.47 | (0.19-1.13) | 0.090 |
| Site of disease (versus pulmonary only) |  |  |  |  |  |  |  |
| Pulmonary plus other site | 0.96 | (0.64-1.43) | 0.843 |  |  |  |  |

**Table S2: Univariate and multivariate logistic regression analyses of factors associated with whether a chest-x-ray/CT scan was performed among Victorian patients with extrapulmonary TB, 2002-2015.**

|  | **Univariate analysis** | | |  | **Multivariate analysis** | | |
| --- | --- | --- | --- | --- | --- | --- | --- |
| **Variable** | **OR** | **(95%CI)** | **P value** |  | **OR** | **(95%CI)** | **P value** |
| Sex: Males versus females | 1.29 | (1.00-1.66) | 0.046 |  | 1.23 | (0.95-1.59) | 0.112 |
| Age, years (versus 0-9 years) |  |  |  |  |  |  |  |
| 10-17 | 1.39 | (0.49-3.98) | 0.538 |  | 1.27 | (0.44-3.65) | 0.661 |
| 18-34 | 1.32 | (0.61-2.87) | 0.477 |  | 1.16 | (0.53-2.53) | 0.711 |
| 35-64 | 1.20 | (0.55-2.61) | 0.652 |  | 1.14 | (0.52-2.50) | 0.741 |
| ≥65 | 0.95 | (0.42-2.15) | 0.899 |  | 0.93 | (0.41-2.11) | 0.858 |
| Year of notification (versus 2002-2005) |  |  |  |  |  |  |  |
| 2006-2011 | 1.31 | (0.98-1.73) | 0.065 |  | 1.26 | (0.95-1.68) | 0.110 |
| 2012-2015 | 2.72 | (1.92-3.84) | <0.001 |  | 2.61 | (1.84-3.71) | <0.001 |
| Healthcare provider |  |  |  |  |  |  |  |
| Private | 1.28 | (0.77-2.13) | 0.333 |  |  |  |  |
| Rural | 1.27 | (0.38-4.20) | 0.699 |  |  |  |  |
| Risk factors considered |  |  |  |  |  |  |  |
| Overseas born | 1.10 | (0.66-1.83) | 0.723 |  |  |  |  |
| Born in a high burden country | 0.96 | (0.74-1.25) | 0.762 |  |  |  |  |
| Household member of close contact with TB | 0.90 | (0.63-1.28) | 0.562 |  |  |  |  |
| Ever resided in an aged care facility | 1.29 | (0.16-10.20) | 0.811 |  |  |  |  |
| History of substance abuse | 1.58 | (0.20-12.25) | 0.664 |  |  |  |  |
| Site of disease (versus lymph node) |  |  |  |  |  |  |  |
| Extrapulmonary, other | 1.11 | (0.86-1.43) | 0.435 |  |  |  |  |

**Table S3: Univariate and multivariate logistic regression analyses of factors associated with whether a smear sputum sample was taken among Victorian TB patients with pulmonary involvement, 2002-2015.**

|  | **Univariate analysis** | | |  | **Multivariate analysis** | | |
| --- | --- | --- | --- | --- | --- | --- | --- |
| **Variable** | **OR** | **(95%CI)** | **P value** |  | **OR** | **(95%CI)** | **P value** |
| Sex: Males versus females | 1.20 | (0.99-1.45) | 0.057 |  | 1.25 | (1.02-1.54) | 0.030 |
| Age, years (versus 0-9 years) |  |  |  |  |  |  |  |
| 10-17 | 12.70 | (6.66-24.21) | <0.001 |  | 14.00 | (7.11-27.54) | <0.001 |
| 18-34 | 22.16 | (13.49-36.41) | <0.001 |  | 25.72 | (14.58-45.37) | <0.001 |
| 35-64 | 18.38 | (11.08-30.51) | <0.001 |  | 22.06 | (12.43-39.13) | <0.001 |
| ≥65 | 17.43 | (10.38-29.26) | <0.001 |  | 19.87 | (11.10-35.56) | <0.001 |
| Year of notification (versus 2002-2005) |  |  |  |  |  |  |  |
| 2006-2011 | 1.38 | (1.06-1.79) | 0.017 |  | 1.42 | (1.07-1.90) | 0.017 |
| 2012-2015 | 0.70 | (0.55-0.90) | 0.004 |  | 0.67 | (0.52-0.88) | 0.003 |
| Healthcare provider |  |  |  |  |  |  |  |
| Private | 0.63 | (0.39-1.02) | 0.062 |  | 0.52 | (0.31-0.86) | 0.011 |
| Rural | 1.05 | (0.58-1.90) | 0.877 |  |  |  |  |
| Risk factors considered |  |  |  |  |  |  |  |
| Overseas born | 2.06 | (1.63-2.61) | <0.001 |  | 0.89 | (0.63-1.26) | 0.510 |
| Born in a high burden country | 1.45 | (1.20-1.74) | <0.001 |  | 1.02 | (0.81-1.29) | 0.842 |
| Household member of close contact with TB | 0.54 | (0.43-0.66) | <0.001 |  | 0.97 | (0.74-1.27) | 0.825 |
| Ever resided in an aged care facility | 0.69 | (0.30-1.58) | 0.382 |  |  |  |  |
| History of substance abuse | 1.05 | (0.57-1.95) | 0.879 |  |  |  |  |
| Result of first chest-x-ray/CT scan | 0.92 | (0.56-1.50) | 0.728 |  |  |  |  |
| Site of disease (versus pulmonary only) |  |  |  |  |  |  |  |
| Pulmonary plus other site | 0.83 | (0.66-1.04) | 0.113 |  | 0.74 | (0.58-0.95) | 0.016 |

**Table S4: Univariate and multivariate logistic regression analyses of factors associated with whether a bronchial wash sample was taken among Victorian TB patients with pulmonary involvement, 2002-2015.**

|  | **Univariate analysis** | | |  | **Multivariate analysis** | | |
| --- | --- | --- | --- | --- | --- | --- | --- |
| **Variable** | **OR** | **(95%CI)** | **P value** |  | **OR** | **(95%CI)** | **P value** |
| Sex: Males versus females | 0.93 | (0.78-1.09) | 0.365 |  |  |  |  |
| Age, years (versus 0-9 years) |  |  |  |  |  |  |  |
| 10-17 | 7.60 | (2.16-26.77) | 0.002 |  | 0.77 | (0.35-1.68) | 0.506 |
| 18-34 | 21.79 | (6.88-68.99) | <0.001 |  | 1.50 | (1.07-2.12) | 0.019 |
| 35-64 | 19.28 | (6.06-61.31) | <0.001 |  | 1.19 | (0.82-1.72) | 0.355 |
| ≥65 | 15.91 | (4.98-50.85) | <0.001 |  | 1.00 | (0.00-0.00) | <0.001 |
| Year of notification (versus 2002-2005) |  |  |  |  |  |  |  |
| 2006-2011 | 6.59 | (4.76-9.13) | <0.001 |  | 11.57 | (6.74-19.85) | <0.001 |
| 2012-2015 | 7.61 | (5.51-10.51) | <0.001 |  | 10.98 | (6.40-18.81) | <0.001 |
| Healthcare provider |  |  |  |  |  |  |  |
| Private | 1.55 | (0.99-2.42) | 0.053 |  | 1.49 | (0.75-2.96) | 0.252 |
| Rural | 0.75 | (0.44-1.30) | 0.308 |  |  |  |  |
| Risk factors considered |  |  |  |  |  |  |  |
| Overseas born | 1.86 | (1.44-2.41) | <0.001 |  | 0.90 | (0.58-1.41) | 0.650 |
| Born in a high burden country | 1.38 | (1.17-1.63) | <0.001 |  | 0.92 | (0.69-1.23) | 0.569 |
| Household member of close contact with TB | 0.74 | (0.60-0.91) | 0.005 |  | 0.58 | (0.37-0.92) | 0.021 |
| Ever resided in an aged care facility | 0.35 | (0.12-1.02) | 0.053 |  | 0.28 | (0.03-2.45) | 0.252 |
| History of substance abuse | 0.61 | (0.34-1.12) | 0.111 |  | 0.92 | (0.36-2.37) | 0.868 |
| Result of first chest-x-ray/CT scan | 1.75 | (1.09-2.81) | 0.020 |  | 2.70 | (1.45-5.03) | 0.002 |
| Positive sputum smear result | 0.14 | (0.11-0.19) | <0.001 |  | 0.12 | (0.09-0.16) | <0.001 |
| Site of disease (versus pulmonary only) |  |  |  |  |  |  |  |
| Pulmonary plus other site | 1.10 | (0.89-1.35) | 0.381 |  |  |  |  |
| Household member of close contact with TB # Born in a high-burden country |  |  |  |  | 2.06 | (1.11-3.79) | 0.021 |

**# Interaction term**

**Table S5: Univariate and multivariate logistic regression analyses of factors associated with whether a smear test was performed among Victorian TB patients with extrapulmonary TB, 2002-2015.**

|  | **Univariate analysis** | | |  | **Multivariate analysis** | | |
| --- | --- | --- | --- | --- | --- | --- | --- |
| **Variable** | **OR** | **(95%CI)** | **P value** |  | **OR** | **(95%CI)** | **P value** |
| Sex: Males versus females | 1.16 | (0.98-1.38) | 0.092 |  | 1.04 | (0.85-1.28) | 0.705 |
| Age, years (versus 0-9 years) |  |  |  |  |  |  |  |
| 10-17 | 3.85 | (1.82-8.14) | <0.001 |  | 3.95 | (1.66-9.39) | 0.002 |
| 18-34 | 4.90 | (2.72-8.83) | <0.001 |  | 4.36 | (2.13-8.92) | <0.001 |
| 35-64 | 3.84 | (2.12-6.95) | <0.001 |  | 3.65 | (1.78-7.49) | <0.001 |
| ≥65 | 2.62 | (1.41-4.86) | 0.002 |  | 2.44 | (1.17-5.08) | 0.017 |
| Year of notification (versus 2002-2005) |  |  |  |  |  |  |  |
| 2006-2011 | 2.18 | (1.76-2.70) | <0.001 |  | 2.65 | (2.05-3.44) | <0.001 |
| 2012-2015 | 2.14 | (1.72-2.67) | <0.001 |  | 2.02 | (1.57-2.60) | <0.001 |
| Healthcare provider |  |  |  |  |  |  |  |
| Private | 0.59 | (0.43-0.80) | 0.001 |  | 0.54 | (0.37-0.77) | 0.001 |
| Rural | 0.88 | (0.43-1.83) | 0.734 |  |  |  |  |
| Risk factors considered |  |  |  |  |  |  |  |
| Overseas born | 1.58 | (1.11-2.24) | 0.012 |  | 0.95 | (0.58-1.55) | 0.841 |
| Born in a high burden country | 1.40 | (1.17-1.67) | <0.001 |  | 1.22 | (0.97-1.53) | 0.083 |
| Household member of close contact with TB | 0.77 | (0.60-0.98) | 0.037 |  | 0.85 | (0.63-1.15) | 0.289 |
| Ever resided in an aged care facility | 5.00 | (0.63-39.52) | 0.127 |  |  | omitted |  |
| History of substance abuse | 1.66 | (0.45-6.16) | 0.447 |  |  |  |  |
| Result of first chest-x-ray/CT scan | 1.20 | (0.98-1.47) | 0.074 |  | 1.26 | (1.01-1.56) | 0.041 |
| Site of disease (versus lymph node) |  |  |  |  |  |  |  |
| Extrapulmonary, other | 1.01 | (0.85-1.21) | 0.889 |  |  |  |  |

**Table S6: Univariate and multivariate logistic regression analyses of factors associated with whether genotypic testing was performed among Victorian TB patients 2002-2015.**

|  | **Univariate analysis** | | |  | **Multivariate analysis** | | |
| --- | --- | --- | --- | --- | --- | --- | --- |
| **Variable** | **OR** | **(95%CI)** | **P value** |  | **OR** | **(95%CI)** | **P value** |
| Sex: Males versus females | 1.03 | (0.92-1.16) | 0.555 |  |  |  |  |
| Age, years (versus 0-9 years) |  |  |  |  |  |  |  |
| 10-17 | 0.82 | (0.49-1.38) | 0.452 |  | 1.18 | (0.64-2.16) | 0.594 |
| 18-34 | 0.67 | (0.44-1.01) | 0.058 |  | 0.92 | (0.55-1.53) | 0.739 |
| 35-64 | 0.64 | (0.42-0.98) | 0.038 |  | 0.95 | (0.57-1.60) | 0.858 |
| ≥65 | 0.69 | (0.44-1.06) | 0.088 |  | 1.08 | (0.64-1.82) | 0.784 |
| Year of notification (versus 2002-2005) |  |  |  |  |  |  |  |
| 2006-2011 | 1.84 | (1.58-2.15) | <0.001 |  | 1.82 | (1.51-2.18) | <0.001 |
| 2012-2015 | 4.19 | (3.59-4.90) | <0.001 |  | 4.29 | (3.58-5.14) | <0.001 |
| Healthcare provider |  |  |  |  |  |  |  |
| Private | 0.58 | (0.45-0.76) | <0.001 |  | 0.65 | (0.47-0.89) | 0.008 |
| Rural | 1.42 | (0.95-2.11) | 0.086 |  | 1.28 | (0.81-2.02) | 0.284 |
| Risk factors considered |  |  |  |  |  |  |  |
| Overseas born | 0.68 | (0.56-0.82) | <0.001 |  | 0.78 | (0.60-1.01) | 0.064 |
| Born in a high burden country | 0.90 | (0.80-1.01) | 0.062 |  | 0.99 | (0.86-1.14) | 0.878 |
| Household member of close contact with TB | 1.04 | (0.89-1.21) | 0.645 |  |  |  |  |
| Ever resided in an aged care facility | 0.85 | (0.45-1.59) | 0.605 |  |  |  |  |
| History of substance abuse | 1.30 | (0.84-2.03) | 0.237 |  | 1.30 | (0.76-2.20) | 0.335 |
| Result of first chest-x-ray/CT scan | 1.41 | (1.24-1.62) | <0.001 |  | 1.47 | (0.82-2.61) | 0.192 |
| Site of disease (versus pulmonary only) |  |  |  |  |  |  |  |
| Pulmonary plus other site | 1.35 | (1.11-1.65) | 0.003 |  | 2.75 | (1.15-6.60) | 0.023 |
| Extrapulmonary, other | 0.59 | (0.51-0.69) | <0.001 |  | 1.06 | (0.59-1.91) | 0.849 |
| Lymph node | 0.99 | (0.84-1.15) | 0.853 |  | 1.37 | (0.75-2.51) | 0.309 |
| Disseminated | 1.26 | (0.98-1.64) | 0.076 |  | 1.48 | (0.59-3.72) | 0.408 |
| Result of first chest-x-ray/CT scan # Site of disease |  |  |  |  |  |  |  |
| Abnormal # Pulmonary plus other site |  |  |  |  | 0.40 | (0.16-0.98) | 0.044 |
| Abnormal # Lymph node |  |  |  |  | 0.52 | (0.27-1.00) | 0.049 |
| Abnormal # Extrapulmonary, other |  |  |  |  | 0.91 | (0.48-1.75) | 0.780 |
| Abnormal # Disseminated |  |  |  |  | 0.94 | (0.35-2.49) | 0.899 |

# Interaction term

**Investigations results (from Table 2)**

**Table S7: Univariate and multivariate logistic regression analyses of factors associated with an abnormal first chest-x-ray/CT scan among Victorian TB patients with pulmonary involvement, 2002-2015.**

|  | **Univariate analysis** | | |  | **Multivariate analysis** | | |
| --- | --- | --- | --- | --- | --- | --- | --- |
| **Variable** | **OR** | **(95%CI)** | **P value** |  | **OR** | **(95%CI)** | **P value** |
| Sex: Males versus females | 1.51 | (1.01-2.24) | 0.042 |  | 1.49 | (0.99-2.24) | 0.056 |
| Age, years (versus 0-9 years) |  |  |  |  |  |  |  |
| 10-17 | 0.43 | (0.11-1.78) | 0.246 |  | 0.70 | (0.16-3.06) | 0.632 |
| 18-34 | 0.75 | (0.23-2.48) | 0.643 |  | 1.21 | (0.34-4.37) | 0.767 |
| 35-64 | 0.48 | (0.15-1.59) | 0.230 |  | 0.75 | (0.21-2.70) | 0.663 |
| ≥65 | 0.71 | (0.20-2.47) | 0.592 |  | 1.04 | (0.28-3.87) | 0.954 |
| Year of notification (versus 2002-2005) |  |  |  |  |  |  |  |
| 2006-2011 | 1.63 | (0.97-2.75) | 0.064 |  | 1.71 | (0.99-2.95) | 0.054 |
| 2012-2015 | 1.48 | (0.91-2.41) | 0.117 |  | 1.57 | (0.94-2.62) | 0.086 |
| Healthcare provider |  |  |  |  |  |  |  |
| Private | 3.18 | (0.44-23.12) | 0.253 |  |  |  |  |
| Rural | 0.39 | (0.16-0.92) | 0.032 |  | 0.32 | (0.13-0.78) | 0.013 |
| Risk factors considered |  |  |  |  |  |  |  |
| Overseas born | 0.56 | (0.28-1.13) | 0.104 |  | 0.60 | (0.28-1.29) | 0.194 |
| Born in a high burden country | 0.93 | (0.62-1.38) | 0.708 |  |  |  |  |
| Household member of close contact with TB | 0.84 | (0.53-1.32) | 0.444 |  |  |  |  |
| Ever resided in an aged care facility |  | omitted |  |  |  |  |  |
| History of substance abuse |  | omitted |  |  |  |  |  |
| Site of disease (versus pulmonary only) |  |  |  |  |  |  |  |
| Pulmonary plus other site | 0.31 | (0.20-0.46) | <0.001 |  | 0.30 | (0.20-0.46) | <0.001 |

**Table S8: Univariate and multivariate logistic regression analyses of factors associated with an abnormal first chest-x-ray/CT scan among Victorian patients with extrapulmonary TB, 2002-2015.**

|  | **Univariate analysis** | | |  | **Multivariate analysis** | | |
| --- | --- | --- | --- | --- | --- | --- | --- |
| **Variable** | **OR** | **(95%CI)** | **P value** |  | **OR** | **(95%CI)** | **P value** |
| Sex: Males versus females | 2.00 | (1.65-2.42) | <0.001 |  | 1.82 | (1.49-2.23) | <0.001 |
| Age, years (versus 0-9 years) |  |  |  |  |  |  |  |
| 10-17 | 0.34 | (0.15-0.77) | 0.010 |  | 0.41 | (0.17-0.95) | 0.037 |
| 18-34 | 0.34 | (0.18-0.65) | 0.001 |  | 0.42 | (0.21-0.85) | 0.016 |
| 35-64 | 0.23 | (0.12-0.44) | <0.001 |  | 0.29 | (0.14-0.59) | 0.001 |
| ≥65 | 0.51 | (0.26-1.01) | 0.053 |  | 0.58 | (0.28-1.21) | 0.147 |
| Year of notification (versus 2002-2005) |  |  |  |  |  |  |  |
| 2006-2011 | 1.18 | (0.93-1.51) | 0.174 |  | 1.14 | (0.88-1.48) | 0.311 |
| 2012-2015 | 1.41 | (1.11-1.80) | 0.005 |  | 1.36 | (1.05-1.76) | 0.019 |
| Healthcare provider |  |  |  |  |  |  |  |
| Private | 0.66 | (0.45-0.95) | 0.024 |  | 0.71 | (0.48-1.04) | 0.080 |
| Rural | 1.44 | (0.68-3.09) | 0.343 |  |  |  |  |
| Risk factors considered |  |  |  |  |  |  |  |
| Overseas born | 0.52 | (0.35-0.77) | 0.001 |  | 0.71 | (0.45-1.14) | 0.156 |
| Born in a high burden country | 0.81 | (0.67-0.98) | 0.033 |  | 0.93 | (0.74-1.15) | 0.489 |
| Household member of close contact with TB | 1.23 | (0.94-1.61) | 0.138 |  | 1.23 | (0.91-1.65) | 0.173 |
| Ever resided in an aged care facility | 1.27 | (0.34-4.75) | 0.720 |  |  |  |  |
| History of substance abuse | 1.59 | (0.46-5.52) | 0.463 |  |  |  |  |
| Site of disease (versus lymph node) |  |  |  |  |  |  |  |
| Extrapulmonary, other | 1.82 | (1.51-2.21) | <0.001 |  | 1.69 | (1.38-2.07) | <0.001 |

**Table S9: Univariate and multivariate logistic regression analyses of factors associated with having a cavitation reported on chest-x-ray among Victorian TB patients with pulmonary involvement, 2002-2015.**

|  | **Univariate analysis** | | |  | **Multivariate analysis** | | |
| --- | --- | --- | --- | --- | --- | --- | --- |
| **Variable** | **OR** | **(95%CI)** | **P value** |  | **OR** | **(95%CI)** | **P value** |
| Sex: Males versus females | 1.12 | (0.90-1.38) | 0.302 |  |  |  |  |
| Age, years (versus 0-9 years) |  |  |  |  |  |  |  |
| 10-17 | 1.65 | (0.92-2.93) | 0.090 |  | 1.65 | (0.91-3.01) | 0.101 |
| 18-34 | 1.56 | (1.14-2.13) | 0.006 |  | 1.77 | (1.27-2.48) | 0.001 |
| 35-64 | 2.13 | (1.53-2.96) | <0.001 |  | 2.38 | (1.68-3.38) | <0.001 |
| ≥65 |  | omitted |  |  |  | omitted |  |
| Year of notification (versus 2002-2005) |  |  |  |  |  |  |  |
| 2006-2011 | 0.82 | (0.62-1.08) | 0.153 |  | 0.80 | (0.60-1.07) | 0.134 |
| 2012-2015 | 0.64 | (0.49-0.84) | 0.001 |  | 0.64 | (0.48-0.86) | 0.003 |
| Healthcare provider |  |  |  |  |  |  |  |
| Private | 0.68 | (0.34-1.34) | 0.262 |  |  |  |  |
| Rural | 1.62 | (0.88-2.98) | 0.119 |  | 1.55 | (0.83-2.88) | 0.169 |
| Risk factors considered |  |  |  |  |  |  |  |
| Overseas born | 1.03 | (0.76-1.38) | 0.862 |  |  |  |  |
| Born in a high burden country | 0.87 | (0.71-1.07) | 0.184 |  | 0.79 | (0.63-0.98) | 0.036 |
| Household member of close contact with TB | 1.07 | (0.83-1.37) | 0.612 |  |  |  |  |
| Ever resided in an aged care facility | 0.70 | (0.24-2.03) | 0.509 |  |  |  |  |
| History of substance abuse | 2.12 | (1.19-3.77) | 0.011 |  | 1.53 | (0.83-2.82) | 0.172 |
| Result of first chest-x-ray/CT scan |  |  |  |  |  |  |  |
| Site of disease (versus pulmonary only) |  |  |  |  |  |  |  |
| Pulmonary plus other site | 0.42 | (0.30-0.58) | <0.001 |  | 0.42 | (0.30-0.59) | <0.001 |

**Table S10: Univariate and multivariate logistic regression analyses of factors associated with a smear positive sputum sample among Victorian TB patients with pulmonary involvement, 2002-2015.**

|  | **Univariate analysis** | | |  | **Multivariate analysis** | | |
| --- | --- | --- | --- | --- | --- | --- | --- |
| **Variable** | **OR** | **(95%CI)** | **P value** |  | **OR** | **(95%CI)** | **P value** |
| Sex: Males versus females | 0.94 | (0.79-1.13) | 0.528 |  |  |  |  |
| Age, years (versus 0-9 years) |  |  |  |  |  |  |  |
| 10-17 | 1.17 | (0.71-1.91) | 0.545 |  | 1.14 | (0.66-1.97) | 0.642 |
| 18-34 | 0.90 | (0.71-1.14) | 0.395 |  | 0.87 | (0.66-1.15) | 0.333 |
| 35-64 | 0.85 | (0.66-1.10) | 0.228 |  | 0.85 | (0.63-1.15) | 0.282 |
| ≥65 |  | omitted |  |  | 1.00 | (0.00-0.00) | <0.001 |
| Year of notification (versus 2002-2005) |  |  |  |  |  |  |  |
| 2006-2011 | 0.74 | (0.59-0.93) | 0.011 |  | 0.78 | (0.59-1.03) | 0.076 |
| 2012-2015 | 0.74 | (0.59-0.94) | 0.013 |  | 0.74 | (0.56-0.98) | 0.033 |
| Healthcare provider |  |  |  |  |  |  |  |
| Private | 0.71 | (0.41-1.24) | 0.225 |  | 0.54 | (0.27-1.05) | 0.070 |
| Rural | 2.48 | (1.41-4.35) | 0.002 |  | 2.69 | (1.41-5.14) | 0.003 |
| Risk factors considered |  |  |  |  |  |  |  |
| Overseas born | 0.86 | (0.66-1.12) | 0.268 |  |  |  |  |
| Born in a high burden country | 0.84 | (0.71-1.01) | 0.061 |  | 0.89 | (0.72-1.08) | 0.239 |
| Household member of close contact with TB | 1.01 | (0.80-1.26) | 0.963 |  |  |  |  |
| Ever resided in an aged care facility | 1.82 | (0.75-4.41) | 0.184 |  | 1.10 | (0.42-2.91) | 0.843 |
| History of substance abuse | 3.14 | (1.72-5.75) | <0.001 |  | 3.13 | (1.59-6.16) | 0.001 |
| Result of first chest-x-ray/CT scan | 3.84 | (2.11-7.00) | <0.001 |  | 3.29 | (1.76-6.15) | <0.001 |
| Site of disease (versus pulmonary only) |  |  |  |  |  |  |  |
| Pulmonary plus other site | 0.31 | (0.24-0.41) | <0.001 |  | 0.30 | (0.22-0.40) | <0.001 |

**Table S11: Univariate and multivariate logistic regression analyses of factors associated with a positive smear test among Victorian extrapulmonary TB patients, 2002-2015.**

|  | **Univariate analysis** | | |  | **Multivariate analysis** | | |
| --- | --- | --- | --- | --- | --- | --- | --- |
| **Variable** | **OR** | **(95%CI)** | **P value** |  | **OR** | **(95%CI)** | **P value** |
| Sex: Males versus females | 0.93 | (0.72-1.20) | 0.575 |  |  |  |  |
| Age, years (versus 0-9 years) |  |  |  |  |  |  |  |
| 10-17 | 2.17 | (0.42-11.30) | 0.359 |  |  |  |  |
| 18-34 | 1.81 | (0.39-8.33) | 0.448 |  |  |  |  |
| 35-64 | 2.10 | (0.45-9.74) | 0.342 |  |  |  |  |
| ≥65 | 2.13 | (0.44-10.18) | 0.345 |  |  |  |  |
| Year of notification (versus 2002-2005) |  |  |  |  |  |  |  |
| 2006-2011 | 0.37 | (0.27-0.51) | <0.001 |  | 0.40 | (0.28-0.59) | <0.001 |
| 2012-2015 | 0.25 | (0.18-0.35) | <0.001 |  | 0.27 | (0.19-0.40) | <0.001 |
| Healthcare provider |  |  |  |  |  |  |  |
| Private | 1.73 | (1.10-2.71) | 0.017 |  | 1.51 | (0.88-2.56) | 0.132 |
| Rural | 0.60 | (0.17-2.12) | 0.43 |  |  |  |  |
| Risk factors considered |  |  |  |  |  |  |  |
| Overseas born | 0.74 | (0.42-1.33) | 0.321 |  |  |  |  |
| Born in a high burden country | 1.43 | (1.08-1.88) | 0.012 |  | 1.48 | (1.08-2.04) | 0.016 |
| Household member of close contact with TB | 1.08 | (0.74-1.57) | 0.688 |  |  | #N/A |  |
| Ever resided in an aged care facility | 1.29 | (0.24-7.08) | 0.769 |  |  | #N/A |  |
| History of substance abuse |  | omitted |  |  |  | #N/A |  |
| Result of first chest-x-ray/CT scan | 0.62 | (0.47-0.83) | 0.001 |  | 0.69 | (0.51-0.94) | 0.018 |
| Site of disease (versus lymph node) |  |  |  |  |  |  |  |
| Extrapulmonary, other | 0.80 | (0.62-1.03) | 0.089 |  | 0.84 | (0.62-1.13) | 0.243 |

**Table S12: Univariate and multivariate logistic regression analyses of factors associated with a positive genotypic test among Victorian TB patients, 2002-2015.**

|  | **Univariate analysis** | | |  | **Multivariate analysis** | | |
| --- | --- | --- | --- | --- | --- | --- | --- |
| **Variable** | **OR** | **(95%CI)** | **P value** |  | **OR** | **(95%CI)** | **P value** |
| Sex: Males versus females | 0.97 | (0.78-1.20) | 0.757 |  |  |  |  |
| Age, years (versus 0-9 years) |  |  |  |  |  |  |  |
| 10-17 | 2.16 | (0.86-5.44) | 0.102 |  | 4.03 | (1.37-11.84) | 0.011 |
| 18-34 | 1.42 | (0.73-2.76) | 0.298 |  | 2.31 | (1.07-5.02) | 0.034 |
| 35-64 | 1.43 | (0.73-2.81) | 0.297 |  | 2.03 | (0.93-4.43) | 0.074 |
| ≥65 | 1.73 | (0.86-3.49) | 0.125 |  | 2.73 | (1.22-6.13) | 0.015 |
| Year of notification (versus 2002-2005) |  |  |  |  |  |  |  |
| 2006-2011 | 1.30 | (0.91-1.85) | 0.154 |  | 1.29 | (0.84-1.99) | 0.252 |
| 2012-2015 | 0.74 | (0.54-1.02) | 0.066 |  | 0.67 | (0.45-0.99) | 0.044 |
| Healthcare provider |  |  |  |  |  |  |  |
| Private | 1.00 | (0.58-1.73) | 0.998 |  |  |  |  |
| Rural | 2.19 | (0.87-5.52) | 0.096 |  | 2.74 | (0.82-9.18) | 0.101 |
| Risk factors considered |  |  |  |  |  |  |  |
| Overseas born | 0.65 | (0.44-0.96) | 0.032 |  | 0.65 | (0.38-1.11) | 0.114 |
| Born in a high burden country | 0.89 | (0.71-1.11) | 0.293 |  | 0.97 | (0.74-1.29) | 0.854 |
| Household member of close contact with TB | 1.02 | (0.76-1.38) | 0.876 |  |  |  |  |
| Ever resided in an aged care facility | 1.53 | (0.35-6.71) | 0.574 |  |  |  |  |
| History of substance abuse | 2.00 | (0.71-5.64) | 0.188 |  | 5.67 | (0.76-42.32) | 0.091 |
| Result of first chest-x-ray/CT scan | 1.35 | (1.05-1.74) | 0.018 |  | 0.73 | (0.53-1.03) | 0.070 |
| Site of disease (versus pulmonary only) |  |  |  |  |  |  |  |
| Pulmonary plus other site | 0.75 | (0.51-1.12) | 0.157 |  | 0.68 | (0.44-1.04) | 0.075 |
| Lymph node | 0.62 | (0.45-0.85) | 0.003 |  | 0.54 | (0.36-0.82) | 0.004 |
| Extrapulmonary, other | 0.28 | (0.21-0.37) | <0.001 |  | 0.21 | (0.15-0.29) | <0.001 |
| Disseminated | 0.47 | (0.30-0.73) | 0.001 |  | 0.43 | (0.26-0.70) | 0.001 |

**Table S13: Univariate and multivariate logistic regression analyses of factors associated with a positive culture among Victorian patients, 2002-2015.**

|  | **Univariate analysis** | | |  | **Multivariate analysis** | | |
| --- | --- | --- | --- | --- | --- | --- | --- |
| **Variable** | **OR** | **(95%CI)** | **P value** |  | **OR** | **(95%CI)** | **P value** |
| Sex: Males versus females | 0.87 | (0.70-1.10) | 0.242 |  | 0.86 | (0.67-1.11) | 0.253 |
| Age, years (versus 0-9 years) |  |  |  |  |  |  |  |
| 10-17 | 3.57 | (1.51-8.44) | 0.004 |  | 3.61 | (1.46-8.95) | 0.006 |
| 18-34 | 4.20 | (2.31-7.66) | <0.001 |  | 4.84 | (2.47-9.47) | <0.001 |
| 35-64 | 2.71 | (1.48-4.95) | 0.001 |  | 3.34 | (1.70-6.55) | <0.001 |
| ≥65 | 3.52 | (1.86-6.63) | <0.001 |  | 4.25 | (2.09-8.61) | <0.001 |
| Year of notification (versus 2002-2005) |  |  |  |  |  |  |  |
| 2006-2011 | 1.57 | (1.17-2.09) | 0.002 |  | 1.24 | (0.88-1.75) | 0.227 |
| 2012-2015 | 1.09 | (0.83-1.43) | 0.540 |  | 0.85 | (0.62-1.18) | 0.330 |
| Healthcare provider |  |  |  |  |  |  |  |
| Private | 0.82 | (0.51-1.30) | 0.398 |  |  |  |  |
| Rural | 2.36 | (0.74-7.50) | 0.146 |  | 2.09 | (0.65-6.74) | 0.215 |
| Risk factors considered |  |  |  |  |  |  |  |
| Overseas born | 1.07 | (0.74-1.55) | 0.712 |  |  |  |  |
| Born in a high burden country | 1.25 | (1.00-1.57) | 0.053 |  | 1.16 | (0.89-1.50) | 0.273 |
| Household member of close contact with TB | 0.98 | (0.72-1.32) | 0.893 |  |  |  |  |
| Ever resided in an aged care facility | 1.01 | (0.31-3.30) | 0.986 |  |  |  |  |
| History of substance abuse | 1.59 | (0.58-4.37) | 0.369 |  |  |  |  |
| Result of first chest-x-ray/CT scan | 1.44 | (1.12-1.85) | 0.004 |  | 0.88 | (0.63-1.24) | 0.470 |
| Site of disease (versus pulmonary only) |  |  |  |  |  |  |  |
| Pulmonary plus other site | 1.30 | (0.81-2.09) | 0.284 |  | 1.33 | (0.80-2.21) | 0.266 |
| Lymph node | 0.65 | (0.49-0.87) | 0.004 |  | 0.54 | (0.37-0.79) | 0.002 |
| Extrapulmonary, other | 0.42 | (0.32-0.56) | <0.001 |  | 0.40 | (0.28-0.57) | <0.001 |
| Disseminated | 0.85 | (0.49-1.46) | 0.552 |  | 0.93 | (0.51-1.71) | 0.818 |

**Table S14: Univariate and multivariate logistic regression analyses of factors associated with a presumptive diagnosis (not confirmed by culture or PCR) among Victorian patients with extrapulmonary TB, 2002-2015.**

|  | **Univariate analysis** | | |  | **Multivariate analysis** | | |
| --- | --- | --- | --- | --- | --- | --- | --- |
| **Variable** | **OR** | **(95%CI)** | **P value** |  | **OR** | **(95%CI)** | **P value** |
| Sex: Males versus females | 0.91 | (0.79-1.05) | 0.216 |  | 0.97 | (0.82-1.15) | 0.711 |
| Age, years (versus 0-9 years) |  |  |  |  |  |  |  |
| 10-17 | 0.18 | (0.12-0.29) | <0.001 |  | 0.15 | (0.09-0.26) | <0.001 |
| 18-34 | 0.08 | (0.06-0.11) | <0.001 |  | 0.06 | (0.04-0.09) | <0.001 |
| 35-64 | 0.13 | (0.09-0.18) | <0.001 |  | 0.09 | (0.06-0.14) | <0.001 |
| ≥65 | 0.10 | (0.07-0.14) | <0.001 |  | 0.07 | (0.04-0.11) | <0.001 |
| Year of notification (versus 2002-2005) |  |  |  |  |  |  |  |
| 2006-2011 | 0.79 | (0.66-0.95) | 0.012 |  | 0.90 | (0.72-1.13) | 0.364 |
| 2012-2015 | 0.86 | (0.72-1.03) | 0.098 |  | 1.01 | (0.82-1.26) | 0.897 |
| Healthcare provider |  |  |  |  |  |  |  |
| Private | 1.14 | (0.84-1.54) | 0.414 |  |  |  |  |
| Rural | 0.35 | (0.16-0.75) | 0.007 |  | 0.45 | (0.20-0.99) | 0.047 |
| Risk factors considered |  |  |  |  |  |  |  |
| Overseas born | 0.62 | (0.50-0.76) | <0.001 |  | 1.19 | (0.85-1.66) | 0.307 |
| Born in a high burden country | 0.69 | (0.60-0.79) | <0.001 |  | 0.84 | (0.70-1.01) | 0.067 |
| Household member of close contact with TB | 1.73 | (1.46-2.05) | <0.001 |  | 1.17 | (0.93-1.48) | 0.169 |
| Ever resided in an aged care facility | 0.49 | (0.17-1.38) | 0.178 |  | 0.74 | (0.25-2.19) | 0.587 |
| History of substance abuse | 0.29 | (0.12-0.73) | 0.008 |  | 0.20 | (0.05-0.82) | 0.026 |
| Result of first chest-x-ray/CT scan | 0.56 | (0.48-0.66) | <0.001 |  | 0.86 | (0.70-1.07) | 0.189 |
| Site of disease (versus pulmonary only) |  |  |  |  |  |  |  |
| Pulmonary plus other site | 0.70 | (0.51-0.97) | 0.035 |  | 0.73 | (0.51-1.05) | 0.089 |
| Lymph node | 1.77 | (1.47-2.13) | <0.001 |  | 1.87 | (1.45-2.43) | <0.001 |
| Extrapulmonary, other | 2.78 | (2.30-3.35) | <0.001 |  | 3.03 | (2.38-3.87) | <0.001 |
| Disseminated | 1.32 | (0.93-1.86) | 0.12 |  | 1.18 | (0.78-1.79) | 0.425 |

**Treatment commencement (from Table 2)**

**Table S15: Univariate and multivariate logistic regression analyses of factors associated with commencing treatment prior to any positive test result or abnormal chest x-ray/CT scan, among Victorian TB patients, 2002-2015.**

|  | **Univariate analysis** | | |  | **Multivariate analysis** | | |
| --- | --- | --- | --- | --- | --- | --- | --- |
| **Variable** | **OR** | **(95%CI)** | **P value** |  | **OR** | **(95%CI)** | **P value** |
| Sex: Males versus females | 0.87 | (0.76-1.00) | 0.047 |  | 1.01 | (0.87-1.17) | 0.908 |
| Age, years (versus 0-9 years) |  |  |  |  |  |  |  |
| 10-17 | 1.45 | (0.85-2.47) | 0.176 |  | 1.40 | (0.78-2.51) | 0.252 |
| 18-34 | 1.39 | (0.91-2.11) | 0.126 |  | 1.28 | (0.78-2.10) | 0.335 |
| 35-64 | 1.35 | (0.88-2.06) | 0.172 |  | 1.12 | (0.68-1.84) | 0.669 |
| ≥65 | 1.21 | (0.78-1.89) | 0.389 |  | 1.24 | (0.74-2.07) | 0.407 |
| Year of notification (versus 2002-2005) |  |  |  |  |  |  |  |
| 2006-2011 | 0.80 | (0.68-0.94) | 0.006 |  | 0.80 | (0.67-0.95) | 0.011 |
| 2012-2015 | 0.31 | (0.26-0.37) | <0.001 |  | 0.31 | (0.25-0.37) | <0.001 |
| Healthcare provider |  |  |  |  |  |  |  |
| Private | 1.57 | (1.18-2.09) | 0.002 |  | 1.10 | (0.80-1.51) | 0.575 |
| Rural | 0.68 | (0.41-1.12) | 0.130 |  | 0.80 | (0.47-1.36) | 0.402 |
| Risk factors considered |  |  |  |  |  |  |  |
| Overseas born | 1.29 | (1.02-1.62) | 0.030 |  | 0.89 | (0.66-1.19) | 0.425 |
| Born in a high burden country | 1.28 | (1.11-1.47) | 0.001 |  | 0.85 | (0.65-1.11) | 0.234 |
| Household member of close contact with TB | 0.88 | (0.73-1.05) | 0.147 |  | 1.01 | (0.82-1.23) | 0.959 |
| Ever resided in an aged care facility | 0.56 | (0.23-1.36) | 0.203 |  | 0.69 | (0.28-1.74) | 0.435 |
| History of substance abuse | 0.82 | (0.47-1.44) | 0.494 |  |  |  |  |
| Site of disease (versus pulmonary only) |  |  |  |  |  |  |  |
| Pulmonary plus other site | 1.59 | (1.24-2.05) | <0.001 |  | 1.19 | (0.78-1.81) | 0.421 |
| Lymph node | 3.50 | (2.93-4.17) | <0.001 |  | 2.63 | (1.93-3.58) | <0.001 |
| Extrapulmonary, other | 3.69 | (3.03-4.47) | <0.001 |  | 2.14 | (1.54-2.98) | <0.001 |
| Disseminated | 2.36 | (1.74-3.19) | <0.001 |  | 1.85 | (1.11-3.07) | 0.018 |
| Born in a high burden country # Site of disease |  |  |  |  |  |  |  |
| Yes # Pulmonary plus other site |  |  |  |  | 1.75 | (1.02-2.99) | 0.041 |
| Yes # Lymph node |  |  |  |  | 1.47 | (1.00-2.17) | 0.05 |
| Yes # Extrapulmonary, other |  |  |  |  | 2.26 | (1.48-3.46) | <0.001 |
| Yes # Disseminated |  |  |  |  | 1.37 | (0.72-2.62) | 0.34 |

**# Interaction term**

**Table S16: Univariate and multivariate logistic regression analyses of factors associated with commencing treatment following abnormal chest x-ray/CT scan, before any other results, among Victorian TB patients with pulmonary involvement, 2002-2015.**

|  | **Univariate analysis** | | |  | **Multivariate analysis** | | |
| --- | --- | --- | --- | --- | --- | --- | --- |
| **Variable** | **OR** | **(95%CI)** | **P value** |  | **OR** | **(95%CI)** | **P value** |
| Sex: Males versus females | 0.98 | (0.82-1.16) | 0.791 |  |  |  |  |
| Age, years (versus 0-9 years) |  |  |  |  |  |  |  |
| 10-17 | 0.58 | (0.31-1.06) | 0.078 |  | 0.50 | (0.26-0.96) | 0.038 |
| 18-34 | 0.31 | (0.20-0.49) | <0.001 |  | 0.33 | (0.20-0.55) | <0.001 |
| 35-64 | 0.25 | (0.16-0.41) | <0.001 |  | 0.28 | (0.17-0.47) | <0.001 |
| ≥65 | 0.23 | (0.14-0.37) | <0.001 |  | 0.22 | (0.13-0.38) | <0.001 |
| Year of notification (versus 2002-2005) |  |  |  |  |  |  |  |
| 2006-2011 | 0.59 | (0.47-0.76) | <0.001 |  | 0.60 | (0.47-0.78) | <0.001 |
| 2012-2015 | 0.24 | (0.19-0.31) | <0.001 |  | 0.23 | (0.18-0.30) | <0.001 |
| Healthcare provider |  |  |  |  |  |  |  |
| Private | 0.42 | (0.24-0.74) | 0.003 |  | 0.43 | (0.23-0.78) | 0.006 |
| Rural | 0.51 | (0.28-0.91) | 0.024 |  | 0.47 | (0.25-0.88) | 0.018 |
| Risk factors considered |  |  |  |  |  |  |  |
| Overseas born | 1.12 | (0.88-1.42) | 0.341 |  |  |  |  |
| Born in a high burden country | 0.94 | (0.79-1.11) | 0.459 |  |  |  |  |
| Household member of close contact with TB | 1.47 | (1.21-1.80) | <0.001 |  | 1.23 | (0.97-1.55) | 0.082 |
| Ever resided in an aged care facility | 1.08 | (0.49-2.38) | 0.850 |  |  |  |  |
| History of substance abuse | 0.38 | (0.20-0.72) | 0.003 |  | 0.40 | (0.21-0.78) | 0.007 |
| Site of disease (versus pulmonary only) |  |  |  |  |  |  |  |
| Pulmonary plus other site | 1.02 | (0.82-1.27) | 0.845 |  |  |  |  |

**Table S17: Univariate and multivariate logistic regression analyses of factors associated with commencing treatment following positive culture, among Victorian TB patients with pulmonary involvement, 2002-2015**

|  | **Univariate analysis** | | |  | **Multivariate analysis** | | |
| --- | --- | --- | --- | --- | --- | --- | --- |
| **Variable** | **OR** | **(95%CI)** | **P value** |  | **OR** | **(95%CI)** | **P value** |
| Sex: Males versus females | 1.16 | (0.92-1.46) | 0.204 |  | 1.17 | (0.89-1.56) | 0.266 |
| Age, years (versus 0-9 years) |  |  |  |  |  |  |  |
| 10-17 | 0.20 | (0.08-0.49) | 0.001 |  | 0.23 | (0.08-0.66) | 0.007 |
| 18-34 | 0.51 | (0.39-0.68) | <0.001 |  | 0.45 | (0.32-0.63) | <0.001 |
| 35-64 | 0.74 | (0.55-1.00) | 0.048 |  | 0.57 | (0.39-0.82) | 0.002 |
| ≥65 |  | omitted |  |  |  | omitted |  |
| Year of notification (versus 2002-2005) |  |  |  |  |  |  |  |
| 2006-2011 | 0.98 | (0.72-1.33) | 0.902 |  |  |  |  |
| 2012-2015 | 1.08 | (0.80-1.46) | 0.606 |  |  |  |  |
| Healthcare provider |  |  |  |  |  |  |  |
| Private | 2.59 | (1.56-4.31) | <0.001 |  | 1.22 | (0.58-2.54) | 0.604 |
| Rural | 1.21 | (0.63-2.33) | 0.570 |  |  |  |  |
| Risk factors considered |  |  |  |  |  |  |  |
| Overseas born | 1.07 | (0.78-1.48) | 0.665 |  |  |  |  |
| Born in a high burden country | 1.00 | (0.80-1.25) | 0.979 |  |  |  |  |
| Household member of close contact with TB | 0.74 | (0.55-0.99) | 0.040 |  |  |  |  |
| Ever resided in an aged care facility | 1.83 | (0.73-4.58) | 0.199 |  |  |  |  |
| History of substance abuse | 1.16 | (0.56-2.39) | 0.684 |  |  |  |  |
| Result of first chest-x-ray/CT scan | 0.81 | (0.47-1.41) | 0.458 |  |  |  |  |
| Positive sputum smear result | 0.24 | (0.17-0.34) | <0.001 |  | 0.20 | (0.14-0.28) | <0.001 |
| Site of disease (versus pulmonary only) |  |  |  |  | 0.51 | (0.35-0.74) | <0.001 |
| Pulmonary plus other site | 0.75 | (0.55-1.02) | 0.065 |  | 1.17 | (0.89-1.56) | 0.266 |

**Table S18: Univariate and multivariate logistic regression analyses of factors associated with commencing treatment following positive culture, regardless of other results, among Victorian patients with extrapulmonary TB, 2002-2015**

|  | **Univariate analysis** | | |  | **Multivariate analysis** | | |
| --- | --- | --- | --- | --- | --- | --- | --- |
| **Variable** | **OR** | **(95%CI)** | **P value** |  | **OR** | **(95%CI)** | **P value** |
| Sex: Males versus females | 0.69 | (0.54-0.87) | 0.001 |  | 0.76 | (0.57-1.00) | 0.052 |
| Age, years (versus 0-9 years) |  |  |  |  |  |  |  |
| 10-17 | 5.05 | (1.08-23.70) | 0.04 |  | 6.94 | (0.83-57.89) | 0.073 |
| 18-34 | 4.04 | (0.97-16.79) | 0.055 |  | 6.54 | (0.88-48.53) | 0.066 |
| 35-64 | 5.55 | (1.33-23.11) | 0.019 |  | 9.07 | (1.22-67.48) | 0.031 |
| ≥65 | 5.75 | (1.35-24.43) | 0.018 |  | 9.35 | (1.23-71.28) | 0.031 |
| Year of notification (versus 2002-2005) |  |  |  |  |  |  |  |
| 2006-2011 | 0.93 | (0.71-1.22) | 0.609 |  | 0.73 | (0.52-1.03) | 0.075 |
| 2012-2015 | 0.69 | (0.51-0.93) | 0.014 |  | 0.72 | (0.51-1.01) | 0.061 |
| Healthcare provider |  |  |  |  |  |  |  |
| Private | 1.49 | (1.02-2.18) | 0.040 |  | 1.15 | (0.72-1.84) | 0.548 |
| Rural | 2.80 | (1.33-5.90) | 0.007 |  | 2.75 | (1.18-6.38) | 0.019 |
| Risk factors considered |  |  |  |  |  |  |  |
| Overseas born | 1.22 | (0.73-2.04) | 0.438 |  |  |  |  |
| Born in a high burden country | 1.08 | (0.85-1.37) | 0.554 |  |  |  |  |
| Household member of close contact with TB | 1.08 | (0.78-1.50) | 0.640 |  |  |  |  |
| Ever resided in an aged care facility | 1.46 | (0.30-7.05) | 0.639 |  |  |  |  |
| History of substance abuse | 2.56 | (0.64-10.29) | 0.185 |  | 3.43 | (0.55-21.59) | 0.188 |
| Result of first chest-x-ray/CT scan | 0.72 | (0.54-0.95) | 0.019 |  | 0.88 | (0.66-1.18) | 0.395 |
| Site of disease (versus lymph node TB) |  |  |  |  |  |  |  |
| Extrapulmonary, other | 0.42 | (0.32-0.54) | <0.001 |  | 0.44 | (0.32-0.59) | <0.001 |

**Treatment regimen and outcome (from Table 2)**

**Table S19: Univariate and multivariate logistic regression analyses of factors associated with commencing on fewer than four first-line medications, among Victorian TB patients, 2002-2015 (Confined to patients with no detected resistance, and to patients that commenced treatment, and began on treatment prior to culture results being available).**

|  | **Univariate analysis** | | |  | **Multivariate analysis** | | |
| --- | --- | --- | --- | --- | --- | --- | --- |
| **Variable** | **OR** | **(95%CI)** | **P value** |  | **OR** | **(95%CI)** | **P value** |
| Sex: Males versus females | 0.78 | (0.63-0.97) | 0.022 |  | 0.77 | (0.60-0.99) | 0.044 |
| Age, years (versus 0-9 years) |  |  |  |  |  |  |  |
| 10-17 | 0.18 | (0.11-0.29) | <0.001 |  | 0.21 | (0.13-0.35) | <0.001 |
| 18-34 | 0.01 | (0.01-0.02) | <0.001 |  | 0.02 | (0.01-0.03) | <0.001 |
| 35-64 | 0.03 | (0.02-0.05) | <0.001 |  | 0.04 | (0.02-0.06) | <0.001 |
| ≥65 | 0.15 | (0.10-0.21) | <0.001 |  | 0.16 | (0.11-0.26) | <0.001 |
| Year of notification (versus 2002-2005) |  |  |  |  |  |  |  |
| 2006-2011 | 0.61 | (0.47-0.78) | <0.001 |  | 0.72 | (0.53-0.97) | 0.032 |
| 2012-2015 | 0.47 | (0.36-0.61) | <0.001 |  | 0.58 | (0.42-0.79) | 0.001 |
| Healthcare provider |  |  |  |  |  |  |  |
| Private | 1.79 | (1.19-2.69) | 0.005 |  | 2.17 | (1.36-3.46) | 0.001 |
| Rural | 1.24 | (0.59-2.59) | 0.575 |  |  |  |  |
| Risk factors considered |  |  |  |  |  |  |  |
| Overseas born | 0.20 | (0.16-0.25) | <0.001 |  | 0.98 | (0.68-1.40) | 0.898 |
| Born in a high burden country | 0.25 | (0.20-0.32) | <0.001 |  | 0.57 | (0.43-0.76) | <0.001 |
| Household member of close contact with TB | 3.06 | (2.44-3.84) | <0.001 |  | 1.38 | (1.00-1.91) | 0.050 |
| Ever resided in an aged care facility | 5.10 | (2.38-10.91) | <0.001 |  | 2.32 | (1.01-5.33) | 0.048 |
| History of substance abuse | 0.35 | (0.09-1.44) | 0.147 |  | 0.19 | (0.02-1.44) | 0.108 |
| Site of disease (versus pulmonary only) |  |  |  |  |  |  |  |
| Pulmonary plus other site | 0.88 | (0.60-1.29) | 0.515 |  | 1.15 | (0.73-1.80) | 0.543 |
| Lymph node | 0.77 | (0.57-1.03) | 0.081 |  | 1.08 | (0.75-1.55) | 0.686 |
| Extrapulmonary, other | 1.18 | (0.89-1.55) | 0.250 |  | 1.71 | (1.23-2.39) | 0.001 |
| Disseminated | 1.21 | (0.78-1.87) | 0.398 |  | 1.19 | (0.70-2.00) | 0.520 |

**Table S20: Univariate and multivariate logistic regression analyses of factors associated with completing treatment among Victorian TB patients, 2002-2015.**

|  | **Univariate analysis** | | |  | **Multivariate analysis** | | |
| --- | --- | --- | --- | --- | --- | --- | --- |
| **Variable** | **OR** | **(95%CI)** | **P value** |  | **OR** | **(95%CI)** | **P value** |
| Sex: Males versus females | 0.75 | (0.52-1.09) | 0.132 |  | 0.79 | (0.54-1.17) | 0.243 |
| Age, years (versus 0-9 years) |  |  |  |  |  |  |  |
| 10-17 | 0.39 | (0.07-2.02) | 0.260 |  | 0.36 | (0.07-1.87) | 0.222 |
| 18-34 | 0.39 | (0.09-1.60) | 0.192 |  | 0.33 | (0.08-1.38) | 0.127 |
| 35-64 | 0.49 | (0.12-2.07) | 0.333 |  | 0.53 | (0.12-2.28) | 0.393 |
| ≥65 | 0.34 | (0.08-1.47) | 0.149 |  | 0.37 | (0.08-1.63) | 0.188 |
| Year of notification (versus 2002-2005) |  |  |  |  |  |  |  |
| 2006-2011 | 2.08 | (1.34-3.21) | 0.001 |  | 2.07 | (1.32-3.25) | 0.002 |
| 2012-2015 | 2.09 | (1.33-3.28) | 0.001 |  | 2.07 | (1.30-3.30) | 0.002 |
| Healthcare provider |  |  |  |  |  |  |  |
| Private | 0.58 | (0.30-1.12) | 0.106 |  | 0.61 | (0.31-1.21) | 0.158 |
| Rural | 1.08 | (0.26-4.46) | 0.913 |  |  |  |  |
| Risk factors considered |  |  |  |  |  |  |  |
| Overseas born | 1.06 | (0.59-1.90) | 0.853 |  |  |  |  |
| Born in a high burden country | 1.43 | (0.99-2.06) | 0.057 |  | 1.47 | (0.99-2.18) | 0.055 |
| Household member of close contact with TB | 0.94 | (0.59-1.49) | 0.780 |  |  |  |  |
| Ever resided in an aged care facility |  | omitted |  |  |  |  |  |
| History of substance abuse | 0.22 | (0.10-0.49) | <0.001 |  | 0.18 | (0.08-0.43) | <0.001 |
| Site of disease (versus pulmonary only) |  |  |  |  |  |  |  |
| Pulmonary plus other site | 0.46 | (0.26-0.83) | 0.010 |  | 0.41 | (0.22-0.74) | 0.003 |
| Lymph node | 0.69 | (0.42-1.12) | 0.129 |  | 0.61 | (0.36-1.02) | 0.060 |
| Extrapulmonary, other | 0.59 | (0.36-0.99) | 0.045 |  | 0.57 | (0.33-0.96) | 0.036 |
| Disseminated | 0.56 | (0.25-1.28) | 0.172 |  | 0.64 | (0.26-1.57) | 0.334 |

**Table S21: Univariate and multivariate logistic regression analyses of factors associated with dying before or during treatment among Victorian TB patients, 2002-2015**

|  | **Univariate analysis** | | |  | **Multivariate analysis** | | |
| --- | --- | --- | --- | --- | --- | --- | --- |
| **Variable** | **OR** | **(95%CI)** | **P value** |  | **OR** | **(95%CI)** | **P value** |
| Sex: Males versus females | 2.06 | (1.54-2.76) | <0.001 |  | 1.49 | (1.05-2.11) | 0.025 |
| Age, years (versus 0-9 years) |  |  |  |  |  |  |  |
| 10-17 | 0.02 | (0.00-0.13) | <0.001 |  | 0.02 | (0.00-0.17) | <0.001 |
| 18-34 | 0.01 | (0.00-0.02) | <0.001 |  | 0.01 | (0.00-0.03) | <0.001 |
| 35-64 | 0.07 | (0.04-0.10) | <0.001 |  | 0.07 | (0.05-0.12) | <0.001 |
| ≥65 |  | omitted |  |  |  | omitted |  |
| Year of notification (versus 2002-2005) |  |  |  |  |  |  |  |
| 2006-2011 | 0.62 | (0.46-0.86) | 0.003 |  | 0.94 | (0.63-1.39) | 0.748 |
| 2012-2015 | 0.54 | (0.39-0.76) | <0.001 |  | 0.79 | (0.52-1.19) | 0.261 |
| Healthcare provider |  |  |  |  |  |  |  |
| Private | 1.57 | (0.94-2.63) | 0.084 |  | 1.17 | (0.62-2.19) | 0.630 |
| Rural | 1.77 | (0.81-3.87) | 0.155 |  | 0.73 | (0.30-1.77) | 0.484 |
| Risk factors considered |  |  |  |  |  |  |  |
| Overseas born | 0.40 | (0.29-0.55) | <0.001 |  | 0.93 | (0.58-1.48) | 0.745 |
| Born in a high burden country | 0.37 | (0.28-0.49) | <0.001 |  | 0.65 | (0.46-0.93) | 0.020 |
| Household member of close contact with TB | 0.50 | (0.32-0.78) | 0.002 |  | 0.74 | (0.45-1.22) | 0.233 |
| Ever resided in an aged care facility | 13.58 | (6.99-26.41) | <0.001 |  | 2.18 | (1.05-4.52) | 0.036 |
| History of substance abuse | 4.09 | (2.22-7.53) | <0.001 |  | 1.75 | (0.71-4.26) | 0.221 |
| Site of disease (versus pulmonary only) |  |  |  |  |  |  |  |
| Pulmonary plus other site | 0.90 | (0.58-1.41) | 0.658 |  | 1.18 | (0.71-1.95) | 0.528 |
| Lymph node | 0.14 | (0.08-0.27) | <0.001 |  | 0.34 | (0.17-0.69) | 0.003 |
| Extrapulmonary, other | 0.70 | (0.49-1.02) | 0.062 |  | 0.72 | (0.47-1.12) | 0.149 |
| Disseminated | 2.34 | (1.54-3.54) | <0.001 |  | 2.74 | (1.62-4.62) | <0.001 |

**Investigations undertaken (from Table 3)**

**Table S22: Results of log rank and Cox proportional hazard model analyses for time period between healthcare presentation and treatment commencement (health system delay) among adult Victorian TB patients with pulmonary involvement, 2002-2015.**

|  | **Univariate analysis** | |  | **Multivariate analysis** | | |
| --- | --- | --- | --- | --- | --- | --- |
| **Variable** | **Log rank** | **p value** |  | **HR** | **(95%CI)** | **p value** |
| Sex: Males versus females | 5.36 | 0.021 |  | 1.19 | (1.07-1.33) | 0.002 |
| Age, years (five groups) | 20.24 | <0.001 |  |  |  |  |
| 0-9 |  |  |  | 1 | - | - |
| 10-17 |  |  |  | 0.25 | (0.12-0.51) | <0.001 |
| 18-34 |  |  |  | 0.26 | (0.13-0.52) | <0.001 |
| 35-64 |  |  |  | 0.22 | (0.11-0.44) | <0.001 |
| ≥65 |  |  |  | 0.21 | (0.10-0.41) | <0.001 |
| Year of notification (three groups) | 1.92 | 0.383 |  |  |  |  |
| Healthcare provider |  |  |  |  |  |  |
| Private | 4.83 | 0.028 |  | 0.81 | (0.58-1.12) | 0.195 |
| Rural | 2.79 | 0.095 |  | 1.26 | (0.91-1.74) | 0.171 |
| Risk factors considered |  |  |  |  |  |  |
| Overseas born | 8.98 | 0.003 |  | 1.21 | (1.00-1.45) | 0.044 |
| Born in a high burden country | 14.54 | <0.001 |  | 1.47 | (1.14-1.88) | 0.003 |
| Household member of close contact with TB | 0.30 | 0.585 |  |  |  |  |
| Ever resided in an aged care facility | 0.87 | 0.351 |  |  |  |  |
| History of substance abuse | 0.25 | 0.615 |  |  |  |  |
| Result of first chest-x-ray/CT scan/CT-scan | 0.25 | 0.617 |  |  |  |  |
| Positive sputum smear result | 25.34 | <0.001 |  |  |  |  |
| Site of disease (two groups) | 2.13 | 0.1443 |  |  |  |  |
| Pulmonary only |  |  |  | 1 | - | - |
| Pulmonary plus other site |  |  |  | 0.82 | (0.66-1.02) | 0.075 |
| Born in a high burden country # Site of disease |  |  |  |  |  |  |
| Yes # Pulmonary only |  |  |  | 0.69 | (0.53-0.91) | 0.010 |
| Yes # Pulmonary plus other site |  |  |  | 1 | - | - |

# = interaction operator

**Table S23: Results of log rank and Cox proportional hazard model analyses for time period between healthcare presentation first chest x-ray/CT scan (diagnostic delay) among adult Victorian TB patients with pulmonary involvement, 2002-2015.**

|  | **Univariate analysis** | |  | **Multivariate analysis** | | |
| --- | --- | --- | --- | --- | --- | --- |
| **Variable** | **Log rank** | **p value** |  | **HR** | **(95%CI)** | **p value** |
| Sex: Males versus females | 5.37 | 0.021 |  | 1.14 | (1.01-1.28) | 0.029 |
| Age, years (five groups) | 5.09 | 0.278 |  |  |  |  |
| Year of notification (three groups) | 8.72 | 0.013 |  |  |  |  |
| 2002-2005 |  |  |  | 1 | - | - |
| 2006-2011 |  |  |  | 1.23 | (1.05-1.45) | 0.011 |
| 2012-2015 |  |  |  | 1.15 | (0.99-1.35) | 0.068 |
| Healthcare provider |  |  |  |  |  |  |
| Private | 1.37 | 0.242 |  | 0.84 | (0.60-1.18) | 0.307 |
| Rural | 3.40 | 0.065 |  | 1.44 | (0.99-2.09) | 0.056 |
| Risk factors considered |  |  |  |  |  |  |
| Overseas born | 4.29 | 0.038 |  | 1.03 | (0.85-1.24) | 0.760 |
| Born in a high burden country | 16.05 | <0.001 |  | 1.21 | (1.07-1.38) | 0.003 |
| Household member of close contact with TB | 0.03 | 0.870 |  |  |  |  |
| Ever resided in an aged care facility | 1.08 | 0.298 |  |  |  |  |
| History of substance abuse | 0.23 | 0.632 |  |  |  |  |
| Site of disease (two groups) | 2.10 | 0.147 |  |  |  |  |
| Pulmonary only |  |  |  | 1 | - | - |
| Pulmonary plus other site |  |  |  | 0.90 | (0.78-1.04) | 0.146 |

**Table S24: Results of log rank and Cox proportional hazard model analyses for time period between abnormal chest x-ray/CT scan and treatment commencement (treatment initiation delay 1) among adult Victorian TB patients with pulmonary involvement, 2002-2015.**

|  | **Univariate analysis** | |  | **Multivariate analysis** | | |
| --- | --- | --- | --- | --- | --- | --- |
| **Variable** | **Log rank** | **p value** |  | **HR** | **(95%CI)** | **p value** |
| Sex: Males versus females | <0.01 | 0.954 |  |  |  |  |
| Age, years (five groups) | 48.21 | <0.001 |  |  |  |  |
| 0-9 |  |  |  | 1 | - | - |
| 10-17 |  |  |  | 1.08 | (0.48-2.44) | 0.856 |
| 18-34 |  |  |  | 0.78 | (0.41-1.49) | 0.459 |
| 35-64 |  |  |  | 0.35 | (0.18-0.69) | 0.002 |
| ≥65 |  |  |  | 0.33 | (0.17-0.64) | 0.001 |
| Year of notification (three groups) | 2.78 | 0.249 |  |  |  |  |
| 2002-2005 |  |  |  | 0.91 | (0.79-1.06) | 0.224 |
| 2006-2011 |  |  |  | 1.06 | (0.92-1.22) | 0.436 |
| 2012-2015 |  |  |  |  |  |  |
| Healthcare provider |  |  |  |  |  |  |
| Private | 4.54 | 0.033 |  | 1.35 | (0.82-2.21) | 0.235 |
| Rural | 0.01 | 0.940 |  |  |  |  |
| Risk factors considered |  |  |  |  |  |  |
| Overseas born | 2.31 | 0.129 |  | 0.37 | (0.08-1.67) | 0.196 |
| Born in a high burden country | 2.31 | 0.129 |  | 1.02 | (0.90-1.15) | 0.803 |
| Household member of close contact with TB | 8.49 | 0.004 |  | 1.01 | (0.88-1.16) | 0.921 |
| Ever resided in an aged care facility | 0.43 |  |  |  |  |  |
| History of substance abuse | 3.71 | 0.054 |  | 1.43 | (1.01-2.01) | 0.043 |
| Result of first chest-x-ray/CT scan |  |  |  |  |  |  |
| Positive sputum smear result | 197.30 | <0.001 |  | 1.67 | (1.28-2.17) | <0.001 |
| Site of disease (two groups) | 1.08 | 0.299 |  |  |  |  |
| Born in a high burden country # Private healthcare provider |  |  |  | 0.41 | (0.20-0.81) | 0.011 |
| Age group # Overseas born |  |  |  |  |  |  |
| 10-17 years # Yes |  |  |  | 1.82 | (0.35-9.43) | 0.475 |
| 18-34 years # Yes |  |  |  | 1.77 | (0.38-8.15) | 0.463 |
| 35-64 years # Yes |  |  |  | 3.71 | (0.80-17.23) | 0.094 |
| >=65years # Yes |  |  |  | 4.72 | (1.01-21.95) | 0.048 |
| Age group # Positive sputum smear result |  |  |  |  |  |  |
| 10-17 years # Yes |  |  |  | 2.15 | (1.14-4.03) | 0.017 |
| 18-34 years # Yes |  |  |  | 1.35 | (1.00-1.83) | 0.051 |
| 35-64 years # Yes |  |  |  | 1.46 | (1.04-2.04) | 0.027 |
| >=65years # Yes |  |  |  | 1 | (omitted) |  |

**Table S25: Results of log rank and Cox proportional hazard model analyses for time period between healthcare presentation and treatment commencement (health system delay) among adult Victorian patients with extrapulmonary TB, 2002-2015.**

|  | **Univariate analysis** | |  | **Multivariate analysis** | | |
| --- | --- | --- | --- | --- | --- | --- |
| **Variable** | **Log rank** | **p value** |  | **HR** | **(95%CI)** | **p value** |
| Sex: Males versus females | 8.91 | 0.003 |  | 1.09 | (0.98-1.21) | 0.120 |
| Age, years (five groups) | 3.97 | 0.410 |  |  |  |  |
| Year of notification (three groups) | 21.61 | <0.001 |  |  |  |  |
| 2002-2005 |  |  |  | 1 | - | - |
| 2006-2011 |  |  |  | 1.07 | (0.94-1.22) | 0.303 |
| 2012-2015 |  |  |  | 0.81 | (0.71-0.92) | 0.001 |
| Healthcare provider |  |  |  |  |  |  |
| Private | 1.16 | 0.282 |  |  |  |  |
| Rural | 0.11 | 0.741 |  |  |  |  |
| Risk factors considered |  |  |  |  |  |  |
| Overseas born | 0.02 | 0.890 |  |  |  |  |
| Born in a high burden country | 0.28 | 0.595 |  |  |  |  |
| Household member of close contact with TB | 6.55 | 0.011 |  | 0.81 | (0.69-0.94) | 0.007 |
| Ever resided in an aged care facility | 3.58 | 0.058 |  | 2.23 | (1.06-4.71) | 0.035 |
| History of substance abuse | 0.00 | 0.950 |  |  |  |  |
| Result of first chest-x-ray/CT scan | 5.67 | 0.017 |  | 0.91 | (0.78-1.06) | 0.230 |
| Site of disease (two groups) | 8.71 | 0.003 |  |  |  |  |
| Lymph node |  |  |  | 1 | - | - |
| Extrapulmonary, other |  |  |  | 1.01 | (0.89-1.15) | 0.853 |
| Result of first chest-x-ray/CT scan # Site of disease |  |  |  |  |  |  |
| Abnormal # Extrapulmonary, other |  |  |  | 1.51 | (1.22-1.88) | <0.001 |

# = interaction operator

**Table S26: Results of log rank and Cox proportional hazard model analyses for time period between healthcare presentation first chest x-ray/CT scan (diagnostic delay) among adult Victorian patients with extrapulmonary TB, 2002-2015.**

|  | **Univariate analysis** | |  | **Multivariate analysis** | | |
| --- | --- | --- | --- | --- | --- | --- |
| **Variable** | **Log rank** | **p value** |  | **HR** | **(95%CI)** | **p value** |
| Sex: Males versus females | 1.91 | 0.167 |  | 1.09 | (0.97-1.23) | 0.163 |
| Age, years (five groups) | 1.57 | 0.814 |  |  |  |  |
| 0-9 |  |  |  |  |  |  |
| 10-17 |  |  |  |  |  |  |
| 18-34 |  |  |  |  |  |  |
| 35-64 |  |  |  |  |  |  |
| ≥65 |  |  |  |  |  |  |
| Year of notification (three groups) | 11.52 | 0.003 |  |  |  |  |
| 2002-2005 |  |  |  | 1 | - | - |
| 2006-2011 |  |  |  | 1.18 | (1.01-1.38) | 0.038 |
| 2012-2015 |  |  |  | 0.92 | (0.79-1.06) | 0.252 |
| Healthcare provider |  |  |  |  |  |  |
| Private | 3.66 | 0.056 |  | 0.79 | (0.63-0.99) | 0.043 |
| Rural | 0.34 | 0.561 |  |  |  |  |
| Risk factors considered |  |  |  |  |  |  |
| Overseas born | <0.01 | 0.959 |  |  |  |  |
| Born in a high burden country | 0.01 | 0.923 |  |  |  |  |
| Household member or close contact with TB | 0.05 | 0.824 |  |  |  |  |
| Ever resided in an aged care facility | 1.20 | 0.273 |  |  |  |  |
| History of substance abuse | 5.18 | 0.023 |  | 2.88 | (1.07-7.74) | 0.036 |
| Site of disease (two groups) ^§^ | 0.07 | 0.794 |  |  |  |  |

^§^ Lymph node and other extrapulmonary

**Table S27: Results of log rank and Cox proportional hazard model analyses for time period between symptom onset and healthcare presentation (patient delay) among adult Victorian TB patients, 2012-2015 (Patient delay could only be considered from 2012-2015 due to small sample numbers in prior years).**

|  | **Univariate analysis** | |  | **Multivariate analysis** | | |
| --- | --- | --- | --- | --- | --- | --- |
| **Variable** | **Log rank** | **p value** |  | **HR** | **(95%CI)** | **p value** |
| Sex: Males versus females | 4.15 | 0.042 |  | 1.10 | (0.96-1.26) | 0.188 |
| Age, years (five groups) | 3.28 | 0.350 |  |  |  |  |
| 0-9 |  |  |  |  |  |  |
| 10-17 |  |  |  |  |  |  |
| 18-34 |  |  |  |  |  |  |
| 35-64 |  |  |  |  |  |  |
| ≥65 |  |  |  |  |  |  |
| Healthcare provider |  |  |  |  |  |  |
| Private | 5.22 | 0.222 |  | 1.36 | (0.98-1.90) | 0.065 |
| Rural | 0.10 | 0.750 |  |  |  |  |
| Risk factors considered |  |  |  |  |  |  |
| Overseas born | 4.52 | 0.034 |  | 0.84 | (0.67-1.04) | 0.103 |
| Born in a high burden country | 0.11 | 0.743 |  |  |  |  |
| Household member of close contact with TB | 1.92 | 0.166 |  | 0.86 | (0.71-1.04) | 0.130 |
| Ever resided in an aged care facility | 0.74 | 0.391 |  |  |  |  |
| History of substance abuse | 1.22 | 0.269 |  |  |  |  |
| Site of disease (versus pulmonary only) | 10.54 | 0.032 |  |  |  |  |
| Pulmonary plus other site |  |  |  | 1.24 | (1.00-1.55) | 0.052 |
| Lymph node |  |  |  | 0.98 | (0.82-1.17) | 0.846 |
| Extrapulmonary, other |  |  |  | 0.88 | (0.73-1.07) | 0.195 |
| Disseminated |  |  |  | 0.98 | (0.72-1.35) | 0.922 |

**Table S28: Results of log rank and Cox proportional hazard model analyses for time period between specimen collection to positive culture result (laboratory delay) among adult Victorian TB patients, 2011-2015 (Lab delay could only be considered from 2011-2015 due to year of notification variable not being proportional. Not done by Site of disease due to small sample sizes).**

|  | **Univariate analysis** | |  | **Multivariate analysis** | | |
| --- | --- | --- | --- | --- | --- | --- |
| **Variable** | **Log rank** | **p value** |  | **HR** | **(95%CI)** | **p value** |
| Sex: Males versus females | 3.34 | 0.068 |  | 1.41 | (0.98-2.01) | 0.062 |
| Age, years (five groups) | 4.12 | 0.391 |  |  |  |  |
| Healthcare provider |  |  |  |  |  |  |
| Private | 1.80 | 0.180 |  | 0.90 | (0.68-1.20) | 0.474 |
| Rural | 1.72 | 0.190 |  | 0.78 | (0.55-1.11) | 0.173 |
| Risk factors considered |  |  |  |  |  |  |
| Overseas born | 3.37 | 0.067 |  | 0.82 | (0.64-1.04) | 0.104 |
| Born in a high burden country | 1.13 | 0.289 |  |  |  |  |
| Household member or close contact with TB | 3.62 | 0.057 |  | 0.90 | (0.73-1.13) | 0.367 |
| Ever resided in an aged care facility | 0.05 | 0.820 |  |  |  |  |
| History of substance abuse | 12.68 | <0.001 |  | 1.55 | (1.07-2.25) | 0.020 |
| Site of disease (five groups) | 54.57 | <0.001 |  |  |  |  |
| Pulmonary only |  |  |  | 1 | - | - |
| Pulmonary plus other site |  |  |  | 0.87 | (0.74-1.03) | 0.107 |
| Lymph node |  |  |  | 0.64 | (0.55-0.74) | <0.001 |
| Extrapulmonary, other |  |  |  | 0.72 | (0.62-0.85) | <0.001 |
| Disseminated |  |  |  | 0.70 | (0.54-0.90) | 0.006 |
| Household member or close contact with TB # Male sex |  |  |  | 1.40 | (1.05-1.87) | 0.024 |
| Overseas born # Male sex |  |  |  | 0.69 | (0.48-1.00) | 0.049 |

# = interaction operator

**Table S29: Results of log rank and Cox proportional hazard model analyses for time period between positive culture result and treatment commencement (treatment initiation delay 2) among adult Victorian TB patients, 2002-2015.**

|  | **Univariate analysis** | |  | **Multivariate analysis** | | |
| --- | --- | --- | --- | --- | --- | --- |
| **Variable** | **Log rank** | **p value** |  | **HR** | **(95%CI)** | **p value** |
| Sex: Males versus females | 2.92 | 0.087 |  | 1.19 | (1.02-1.38) | 0.023 |
| Age, years (five groups) | 8.80 | 0.066 |  |  |  |  |
| 0-9 |  |  |  | 1.04 | (0.30-3.55) | 0.955 |
| 10-17 |  |  |  | 0.88 | (0.28-2.75) | 0.822 |
| 18-34 |  |  |  | 0.74 | (0.23-2.31) | 0.600 |
| 35-64 |  |  |  | 0.69 | (0.22-2.18) | 0.532 |
| ≥65 |  |  |  |  |  |  |
| Year of notification (three groups) | 26.78 | <0.001 |  | 1.08 | (0.90-1.31) | 0.406 |
| 2002-2005 |  |  |  | 0.70 | (0.58-0.85) | <0.001 |
| 2006-2011 |  |  |  | 1.19 | (1.02-1.38) | 0.023 |
| 2012-2015 |  |  |  |  |  |  |
| Healthcare provider |  |  |  |  |  |  |
| Private | 0.37 | 0.543 |  |  |  |  |
| Rural | 0.07 | 0.784 |  |  |  |  |
| Risk factors considered |  |  |  |  |  |  |
| Overseas born | 0.11 | 0.741 |  |  |  |  |
| Born in a high burden country | 0.67 | 0.415 |  |  |  |  |
| Household member or close contact with TB | <0.01 | 0.974 |  |  |  |  |
| Ever resided in an aged care facility | 0.23 | 0.634 |  |  |  |  |
| History of substance abuse | 0.22 | 0.636 |  |  |  |  |
| Result of first chest-x-ray | 0.75 | 0.387 |  |  |  |  |
| Site of disease (five groups) | 3.05 | 0.549 |  |  |  |  |
